# The safety and efficacy of chimeric antigen receptor-T cell therapy in solid malignancies: a systematic review and meta-analysis

**DOI:** 10.3389/fimmu.2026.1957437

**Published:** 2026-09-24

**Authors:** Kunpeng Wang, Lian Li, Zhangneng Yu, Dong Li, Jinting Jiang, Zhicheng Guo, Liang Wang, Hua Zhang, Liangliang Xu, Ming Zhang

**Affiliations:** 1 Division of Liver Surgery, Department of General Surgery, West China Hospital, Sichuan University, Chengdu, China; 2 Department of Cardiovascular Surgery, West China Hospital, Sichuan University, Chengdu, China; 3 Key Laboratory of Birth Defects and Related Diseases of Women and Children of the Ministry of Education (MOE), West China Second University Hospital, Sichuan University, Chengdu, China; 4 Key Laboratory of Chronobiology, National Health Commission of China, Sichuan University, Chengdu, China

**Keywords:** adverse events, CAR-T therapy, efficacy, immunotherapy, meta-analysis, solid tumors

## Abstract

**Introduction:**

Chimeric antigen receptor (CAR)-T cell therapy has achieved remarkable success in hematologic malignancies, prompting its exploration in solid tumors. This systematic review and meta-analysis aimed to evaluate the safety and feasibility of CAR-T therapy for solid tumor treatment.

**Methods:**

We systematically searched PubMed, Cochrane Library, Embase, and Web of Science from database inception to June 1, 2026, for clinical studies evaluating CAR-T therapy in solid tumors. Primary outcomes included the incidence of any-grade and grade ≥3 adverse events (AEs); secondary outcomes were the overall response rate (ORR) and disease control rate (DCR). This review adhered to PRISMA guidelines and was registered with PROSPERO (CRD42024549048).

**Results:**

52 studies with 712 patients were enrolled in our study. Lymphopenia (70%, grade≥3: 48%) was the most common adverse event. The pooled ORR and DCR were 13% (95%CI: 11-16%) and 63% (95%CI: 59-67%), respectively. Subgroup analysis indicated that the incidence of hematologic toxicity was significantly higher in patients who had received lymphodepletion, and intravenous delivery was associated with a higher risk of several AEs. Patients who had undergone lymphodepletion (LD) achieved higher ORR (p = 0.0002) and DCR (p = 0.0083). In comparison to regional delivery, intravenous infusion was associated with higher ORR (p = 0.0345).

**Conclusion:**

CAR-T therapy demonstrates an acceptable safety profile for solid tumors. While lymphodepletion and intravenous delivery correlate with higher ORR and DCR, they also increase adverse event risks. Balancing therapeutic benefits and safety concerns therefore remains a critical consideration for clinical practice.

## 1 Introduction

Inspired by the promising outcomes of CAR-T cell therapy in hematological malignancies (1), clinical trials of CAR-T cells against solid tumors are currently underway worldwide (2). CAR-T cells are usually autologous T cells collected by leukapheresis and genetically modified to express a CAR, which binds to tumor antigens. Following ex vivo expansion, the engineered T cells are then reinfused to recognize and destroy tumor cells (3). First-generation CAR-T cells lack a co-stimulation signal domain, and their limited anti-tumor activity ultimately results in T-cell exhaustion and apoptosis (4). Subsequently, second-generation ones integrate a costimulatory domain into the CD3ζ chain (e.g., CD28 or 4-1BB [CD137]), which enhances T-cell activation and effector functions (5). The third-generation CARs are equipped with multiple costimulatory signaling domains, which further enhance their anti-tumor effects (6). Nowadays, the fourth-generation CARs are capable of functional cytokine expression, which augments the potency and persistence of CAR-T cells, showing greater promise in the preclinical tests (7). However, CAR-T cell therapy presents specific side effects, such as cytokine release syndrome (CRS) and immune effector cell-associated neurotoxicity syndrome (ICANS), which pose challenges to treatment safety, patient tolerability, and clinical management (8).

We identified two meta-analyses that are relevant to the topic of our investigation. One study focused on the safety and efficacy of treatments for digestive system tumors (9), while another only explored the efficacy of therapies for solid tumors (10). Due to the scarcity of included studies, both reports led to unreliable and divergent conclusions. Therefore, we summarized the latest clinical studies, further details of individual participant data were collected to investigate the potential factors that are associated with safety and efficacy, hoping to guide clinical practice, and make recommendations for future studies.

## 2 Materials and methods

This study was conducted in accordance with Preferred Reporting Items for Systematic Reviews and Meta-analyses guidelines [PRISMA (11)] (**Supplementary Table S3**) and was registered with PROSPERO (CRD42024549048).

### 2.1 Search strategy and selection criteria

A comprehensive systematic search was conducted across PubMed, the Cochrane Library, Embase, and Web of Science (**Figure 1**, **Supplementary Table S1**). The search spanned from the inception of these databases up to June 1, 2026, aiming to identify relevant clinical trials investigating CAR-T therapy for solid tumors.

**Figure 1:**
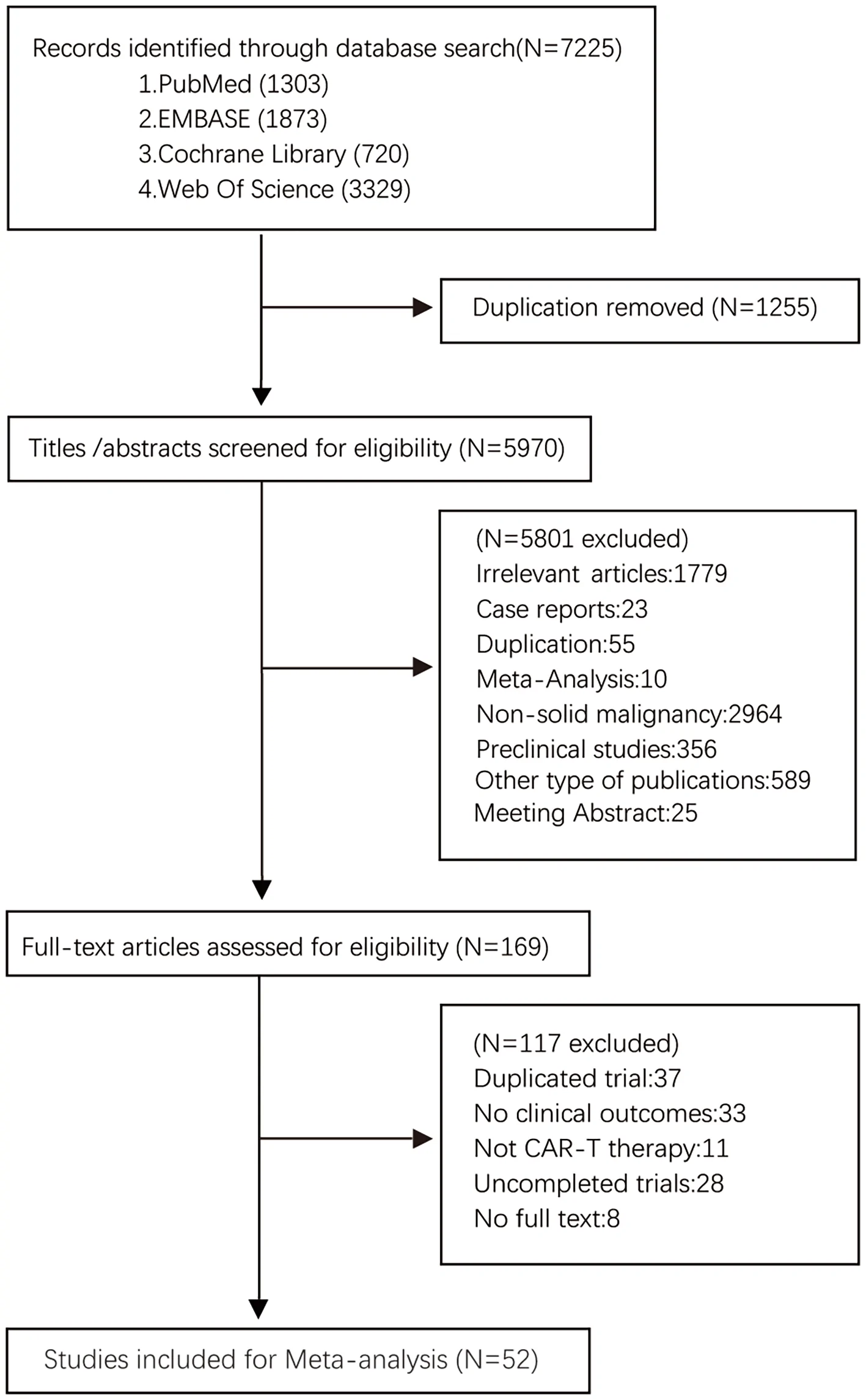
PRISMA flowgram of study selection process. This flow diagram depicts the step-by-step process for study selection (inclusion and exclusion criteria), which was constructed in adherence to the Preferred Reporting Items for Systematic Reviews and Meta-Analyses (PRISMA) guidelines.

Inclusion criteria were:

1. Completed clinical trials involving CAR-T cell therapy in patients with solid tumors;
2. Research articles provided clear reports of the treatment outcomes.

Exclusion criteria were:

1. Duplicated published or outdated studies;
2. Non-English-language articles;
3. Literatures other than research articles, such as meeting abstracts, editorials, comments, reviews, case reports, letters, and meta-analyses;
4. Clinical trials with fewer than 3 participants.

### 2.2 Data extraction

The following data were extracted by from each included study: first author, publication year, country, trial information, target tumor antigen, CAR structure, diagnosis, combination treatment, lymphodepletion, delivery route, dose, transfection method, and outcomes. The primary outcome was safety, which included the incidence of any-grade and grade ≥3 adverse events (AEs). The secondary outcomes comprised the overall response rate (ORR) and disease control rate (DCR). ORR was defined as the sum of complete response (CR) and partial response (PR), while DCR was defined as the sum of CR, PR, and stable disease (SD). If more detailed individual patient data (e.g., survival data) were available in the **Supplementary Materials**, these data were extracted for further analysis.

### 2.3 Data analysis

Subgroup analyses were applied to investigate the factor related to AEs and therapeutic efficacy. Sensitivity analyses were conducted by altering the methods of rate transformation and incorporating individual participant data to evaluate the robustness of the synthesized results.

### 2.4 Quality assessment

The methodological quality of the studies was assessed using MINORS (methodological index for non-randomized studies) which contains 12 items, each item was scored from 0 to 2; 0 indicating that it was not reported, 1 indicating inadequate reporting, and 2 indicating that it was reported adequately (12). The first eight items are specifically for non-comparative studies, thus used to judge the quality of included studies. Publication bias was examined by the visual inspection of funnel plots and confirmed by Egger and Begg tests (13, 14). In this study, the literature search, selection, data extraction, and quality assessment were conducted independently by two authors, and discrepancies were resolved through discussion with a third author when necessary.

### 2.4 Statistical analysis

The Cochrane Q-test and I² statistic were used to assess interstudy heterogeneity, with significant heterogeneity indicated by an I² > 50% and p < 0.1. Both fixed- and random-effects models were employed in this study. Pooled proportions with 95% confidence intervals (CIs) were calculated using the meta package in R. The pooled incidence proportion was estimated using a generalized linear mixed-effects model (GLMM) with a logit transformation. Using the extracted individual patient survival data, survival analysis was performed via the Kaplan–Meier method, and group differences were compared using the log-rank test (R survival and survminer packages). All statistical analyses were executed using R version 4.1.2 (R Foundation for Statistical Computing). A two-sided p < 0.05 was deemed statistically significant.

## 3 Results

### 3.1 Search results

As illustrated in the PRISMA flow diagram (**Figure 1**), 7225 relevant records were initially retrieved. After removing 1255 duplicate records, 5970 potentially eligible records underwent inclusion screening. Based on title and abstract assessment, 5801 studies were excluded, leaving 169 studies for full-text review. After full-text evaluation, ultimately 52 relevant publications involving 712 patients were included in this study.

52 studies were evaluated for methodological quality using a total score of 16 points (8 items rated, 2 points per item), with an average score of 11. Ten studies scored 8–10 points, 36 studies scored 12 points, and four articles scored 13–14 points (**Supplementary Table 2**) (15–17).

### 3.2 Baseline characteristic

The baseline information of studies included in meta-analysis is illustrated in **Table 1**, most studies (46 studies, 88%) were phase I clinical trials (19, 24–26, 31, 34, 39, 41, 46, 48, 49, 53, 55, 58), with sample sizes varying between 3 and 58 participants (22, 43). Approximately half of the studies were published within the past three years, including 4 studies in 2026 (21, 54, 60, 63), 4 studies in 2025 (31, 38, 42, 50), 12 in 2024, and 8 in 2023. 18(37%) and 29 (56%) studies were conducted in China and the United States, respectively. Glioma was the most frequently studied cancer type, with 9 studies, followed by neuroblastoma (5 studies), GD2, EGFR and Mesothelin were the three most commonly targeted antigen. Regarding CAR constructs, with 5 (11%) studies employing first-generation CAR-T cells, 38 (73%) studies utilizing second-generation CAR-T cells, 5 (10%) employing third-generation CAR-T cells, and 2 (4%) utilizing fourth-generation CAR-T cells (45, 61). Notably, in terms of second-generation CAR constructs, 28 (54%) studies used 4-1BB co-stimulated CAR constructs, 10 (20%) studies used CD28 co-stimulated CAR. 32 (61%) studies employed the lymphodepletion conditioning regimen, which typically uses cyclophosphamide (CTX), either alone or in combination with Fludarabine (Flu). Five (10%) studies utilized CAR-T therapy in combination with other antitumor treatment regimens (17, 18, 36, 45, 52). The most common route of administration was intravenous infusion (37 studies, 71%), whereas local administration [intratumoral (30), intraventricular, intraperitoneal (24), or hepatic artery infusions (51)] were tested in 16 studies (26%). 29 studies (56%) used lentiviral transfection of T cells, 16 (31%) used retroviruses, and 6 (12%) used electroporation to introduce CAR constructs into T cells.

**Table 1:** Characteristics of studies included in meta-analysis.

Study(year)	Country	Tumor antigen	Generation	Diagnosis	Tumor type	Trial phase	Delivery	Lymphodepletion	Combination treatment	Dose	Transfection	Participants	CR	PR	SD	PD	IPD
Andras Heczey (2017) (18)	US	GD2	3rd	Neuroblastoma	NB	Phase I	iv	CTX+FLU	N	Multiple	retroviral	11	0	0	5	6	Yes
Andrew R. Haas (2019) (19)	US	Mesothelin	2nd	MPM,Ovarian carcinoma,PDAC	Solid tumors	Phase I	iv	CTX	N	Single	lentiviral	15	0	0	11	4	No
Changsong Qi (2024) (20)	CN	CLDN18.2	2nd	Pancreatic carcinoma	PC	Phase Ib	iv	FLU/CTX+nab-PTX	N	Multiple	lentiviral	24	0	4	13	7	Yes
Chengcheng Zhang(2017) (21)	CN	CEA	2nd	Colorectal cancer	CRC	Phase I	iv	CTX+FLU	N	Multiple	lentiviral	9	0	0	7	2	Yes
Christine E. Brown (2024) (22)	US	IL-13Rα2	2nd	Glioma	Glioma	Phase I	regional	N	N	Multiple	lentiviral	58	1	2	29	26	No
Crystal U. Louis (2011) (23)	US	GD2	1st	Neuroblastoma	NB	Phase I	iv	N	N	Single	retroviral	11	3	1	4	1	Yes
Dan Li (2023) (24)	CN	EpCAM	2nd	Epithelial tumors	Solid tumors	Phase I	iv	CTX+FLU	N	Single	lentiviral	11	0	2	5	4	No
Donald M. O’Rourke(2017) (25)	US	EGFRvIII	2nd	Glioblastoma	Glioma	Phase I	iv	N	N	Single	lentiviral	10	0	0	9	1	No
Donghua Shi(2020) (26)	CN	GPC3	2nd	Hepatocellular Carcinoma	HCC	NA	iv	CTX+FLU	N	Single	lentiviral	9	0	2	2	5	Yes
F. Del Bufalo (2023) (15)	ITALY	GD2	3rd	Neuroblastoma	NB	Phase I,II	iv or regional:intraperitoneally	CTX+FLU	N	Multiple	retroviral	27	9	8	5	5	No
Fiona C. Thistlethwaite (2017) (27)	UK	CEACAM5	1st	Caecum,Gastro-oesophageal junction cancer,Stomach,Colon Cancer	CRC	Phase I	iv	FLU/CTX+FLU	N	Multiple	retroviral	14	0	0	7	7	No
Frank Y. Lin, MD ( 2024) (28)	US	GD2	2nd	CNS tumors	Solid tumors	Phase I	iv	CTX+FLU	N	Multiple	retroviral	11	0	2	5	4	No
Gregory L. Beatty(2018) (29)	US	Mesothelin	2nd	Pancreatic carcinoma	PC	Phase I	iv	N	N	Single	electroporation	6	0	0	2	4	No
Hanren Dai (2020) (26)	CN	CD133	2nd	Hepatocellular Carcinoma	HCC	Phase II	iv	N	N	Multiple	lentiviral	21	0	1	14	6	No
Julia Tchou (2017) (30)	US	cMET	2nd	Breast cancer	BC	Phase 0	regional:intratumoral	N	N	Single	electroporation	6	0	0	1	5	No
Juliane Gust(2025) (31)	US	EGFR	2nd	CNS tumors	Glioma	Phase I	intracranial	N	N	Multiple	lentiviral	4	0	0	1	3	No
Julie R Park (2007) (32)	US	L1-CAM	1st	Neuroblastoma	NB	Phase I	iv	N	N	Multiple	electroporation	6	0	1	0	5	Yes
Kaichao Feng (2016) (33)	CN	EGFR	2nd	NSCLC	NSCLC	Phase I	iv	N	N	Single	lentiviral	11	0	2	5	4	Yes
Kaichao Feng(2017) (34)	CN	HER2	2nd	Biliary tract cancers (BTCs) and pancreatic cancers (PCs)	Solid tumors	Phase I	iv	CTX+nab-PTX	N	Single	lentiviral	11	0	1	5	5	No
Lihua Yu (2022) (35)	CN	GD2	3rd	Neuroblastoma	NB	Phase I	iv	CTX+FLU	N	Single	lentiviral	10	0	0	6	4	Yes
Mackensen.A(2023) (36)	GER	CLDN6	2nd	CLDN6+solid tumors: germ cell tumor, Epithelial ovarian cancer	Mixed Solid tumors	Phase I,II	iv	CTX+FLU	RNA: Vaccine or not	Single	retroviral	21	1	6	7	7	No
Meenakshi Hegde (2024) (37)	US	HER2	2nd	Sarcoma	Sarcoma	Phase I	iv	CTX+FLU	N	Single	retroviral	14	3	0	4	7	Yes
Michael E. Barish (2025) (38)	US	Chlorotoxin	2nd	Phase I	Glioblastoma	Glioma	regional:intracavity/intratumoral	N	N	Multiple	lentiviral	4	0	0	3	1	No
Nabil Ahmed(2017) (39)	US	HER2	2nd	Glioblastoma	Glioma	Phase I	iv	N	Pembrolizumab or not	Single	retroviral	16	0	1	7	8	Yes
Naifei Chen(2024) (40)	CN	GCC	2nd	Colorectal cancer	CRC	Phase I	iv	CTX+FLU	N	Single	lentiviral	15	0	6	5	4	No
Navin Pinto(2024) (41)	US	B7-H3	2nd	Solid Tumors	Solid tumors	Phase I	iv	CTX+FLU	N	Multiple	lentiviral	11	0	0	3	6	No
Nicholas A Vitanza (2025) (42)	US	B7-H3	2nd	DIPG	Glioma	Phase I and Phase I	intraerebroventricular	N	N	Multiple	lentiviral	18	0	1	15	2	No
Nicholas A. Vitanza(2023) (43)	US	B7-H3	2nd	DIPG	Glioma	Phase I	regional:Intraventricular	N	N	Multiple	lentiviral	3	0	0	1	2	Yes
Payal D. Shah (2023) (44)	US	cMET	2nd	TNBC	BC	Phase I	iv	N	N	Multiple	electroporation	7	0	0	4	3	Yes
Prasad S. Adusumilli (2021) (17)	US	Mesothelin	2nd	MPM,Lung cancer,Metastatic breast cancer	Solid tumors	Phase I	regional:intrapleural	CTX	Pembrolizumab,Selective Internal Radiation Therapy	Multiple	retroviral	16	0	2	9	5	No
Qihan Fu (2023) (45)	CN	GPC3	4th	Hepatocellular Carcinoma	HCC	Phase I	iv	CTX+FLU	Sorafenib/regorafenib or not	Single	lentiviral	6	0	1	2	3	No
Richard P. Junghans(2016) (46)	US	PSMA	1st	Prostate cancer	Prostate cancer	Phase I	iv	CTX+FLU	CTX+cisplatin+pemetrexed/docetaxel or not	Single	retroviral	5	0	2	NA	NA	No
Sophie Papa (47)	UK	ErbB	2nd	Head and neck squamous cell carcinoma	Solid tumors	Phase I	regional:intratumoral	N	N	Single	retroviral	15	0	0	9	6	No
Stein, M. N.(2024) (48)	US	PSCA	1st	metastatic pancreatic (mPDAC) or castration-resistant prostate cancer(mCRPC)	Solid tumors	Phase I	iv	CTX+FLU	N	Single	retroviral	26	0	2	16	8	No
Stephanie L. Goff (2019) (49)	US	EGFRvIII	3rd	Glioblastoma	Glioma	Phase I	iv	CTX+FLU	N	Single	retroviral	17	0	0	NA	NA	Yes
Stephen J. Bagley (2024) (16)	US	EGFR and IL13Rα2	2nd	Glioblastoma	Glioma	Phase I	intrathecal	N	N	Multiple	lentiviral	6	0	0	NA	NA	No
Stephen J. Bagley (2025) (50)	US	EGFR and IL13Rα2	2nd	Phase I	Glioblastoma	Glioma	regional:Intraventricular	N	N	Multiple	lentiviral	6	0	0	NA	NA	No
Steven C. Katz (2015) (51)	US	CEA	2nd	Liver metastases	Liver metastasis	Phase I	regional:hepatic artery infusions	N	N	Multiple	retroviral	6	0	0	1	5	Yes
Steven C. Katz(2020) (52)	US	CEA	2nd	Liver metastasis	Liver metastasis	Phase Ib	regional:hepatic artery infusions	N	N	Multiple	retroviral	6	0	0	2	4	Yes
Sumanta K. Pal (2024) (53)	US	CD70	NA	Clear cell renal cell carcinoma	Solid tumors	Phase I	iv	CTX+FLU	N	Multiple	NA	16	1	0	12	3	No
Susan Slovin (2026) (54)	US	PSMA	2nd	Phase I	Prostate cancer	Prostate cancer	iv	CTX+FLU	N	Single	electroporation	33	0	1	20	12	No
Tanya B. Dorff (2024) (55)	US	PSCA	2nd	Prostate cancer	Prostate cancer	Phase I	iv	CTX+FLU or not	N	Single	lentiviral	14	0	0	7	0	No
Tessa Gargett (2024) (56)	AUS	GD2	3rd	Melanoma,fibromyxoid sarcoma,CRC	Sarcoma	Phase I	iv	CTX+FLU	N	Single	retroviral	12	0	5	2	5	Yes
Tongpeng Xu (2024) (57)	CN	Mesothelin	2nd	Ovarian cancer and mesothelioma	Solid tumors	Phase I	iv	CTX+FLU	N	Single	lentiviral	6	0	2	4	0	No
Vivek Narayan (2022) (58)	US	PSMA	2nd	Prostate cancer	Prostate cancer	Phase I	iv	CTX+FLU	N	Single	lentiviral	13	0	0	5	8	No
Yajun Zhang (2021) (59)	CN	EGFR	2nd	NSCLC	NSCLC	Phase I	iv	N	N	Multiple	electroporation	9	0	1	6	2	No
Yang Gao (2026) (60)	CN	CEA	2nd	Phase I	Solid tumors	Mixed Solid tumors	iv or regional:intraperitoneally	CTX+FLU	N	Multiple	lentiviral	42	0	6	25	11	No
Yang Liu (2020) (61)	CN	EGFR	2nd	Pancreatic carcinoma	PC	Phase I	iv	CTX+nab-PTX	N	Multiple	lentiviral	14	0	4	8	2	No
Yelei Guo (62)	CN	EGFR	2nd	Biliary Tract Cancers	Solid tumors	Phase I	iv	CTX+nab-PTX	N	Multiple	lentiviral	17	1	0	10	NA	No
Yongqiang Yang (2026) (63)	CN	GPC3	NA	Phase I	Hepatocellular Carcinoma	HCC	iv	CTX+FLU	N	Single	lentiviral	6	0	0	2	4	No
Zhuohao Liu (2023) (61)	CN	GD2	4th	Glioblastoma	Glioma	Phase I	iv+regional:intracavitary	CTX+FLU	N	Multiple	lentiviral	8	0	4	1	3	No

CN, China; CNS, central nervous system; CR, complete response; CRC, colorectal cancer; CTX, cyclophosphamide; DIPG, diffuse intrinsic pontine glioma; FLU, fludarabine; HCC, hepatocellular carcinoma; IPD, individual participant data; iv, intravenous; MPM, malignant pleural mesothelioma; NA, not available; nab-PTX, nanoparticle albumin-bound paclitaxel; NSCLC, non-small cell lung cancer; PD, progressive disease; PR, partial response; SD, stable disease; US, United States.

### 3.3 Safety and toxicity

CAR-T cell therapy is inevitably accompanied by various adverse events. The most common AEs were lymphopenia (grade-any: 70%, grade≥3: 48%, **Figure 2B**), neutropenia (62%, grade≥3: 41%, **Figure 2D**), and fever (62%, grade≥3: 6%, **Figure 2A**), followed by CRA (53%, grade≥3: 1%, **Supplementary Figure S8G**), anemia (52%, grade≥3: 10%, **Supplementary Figure S8A**) and thrombocytopenia (46%, grade≥3: 19%, **Figure 2E**). Less frequently reported were neurotoxicity (42%, grade≥3: 5%, **Supplementary Figure S8B**), leukopenia (42%, grade≥3: 21%, **Figure 2C**), nausea (33%, grade≥3: 0%, **Supplementary Figure S8D**), vomiting (30%, grade≥3: 1%, **Supplementary Figure S8E**), respiratory symptoms (30%, grade≥3: 4%, **Supplementary Figure S8C**), and dermatological reactions (21%, grade≥3: 1%, **Supplementary Figure S8F**).

**Figure 2:**
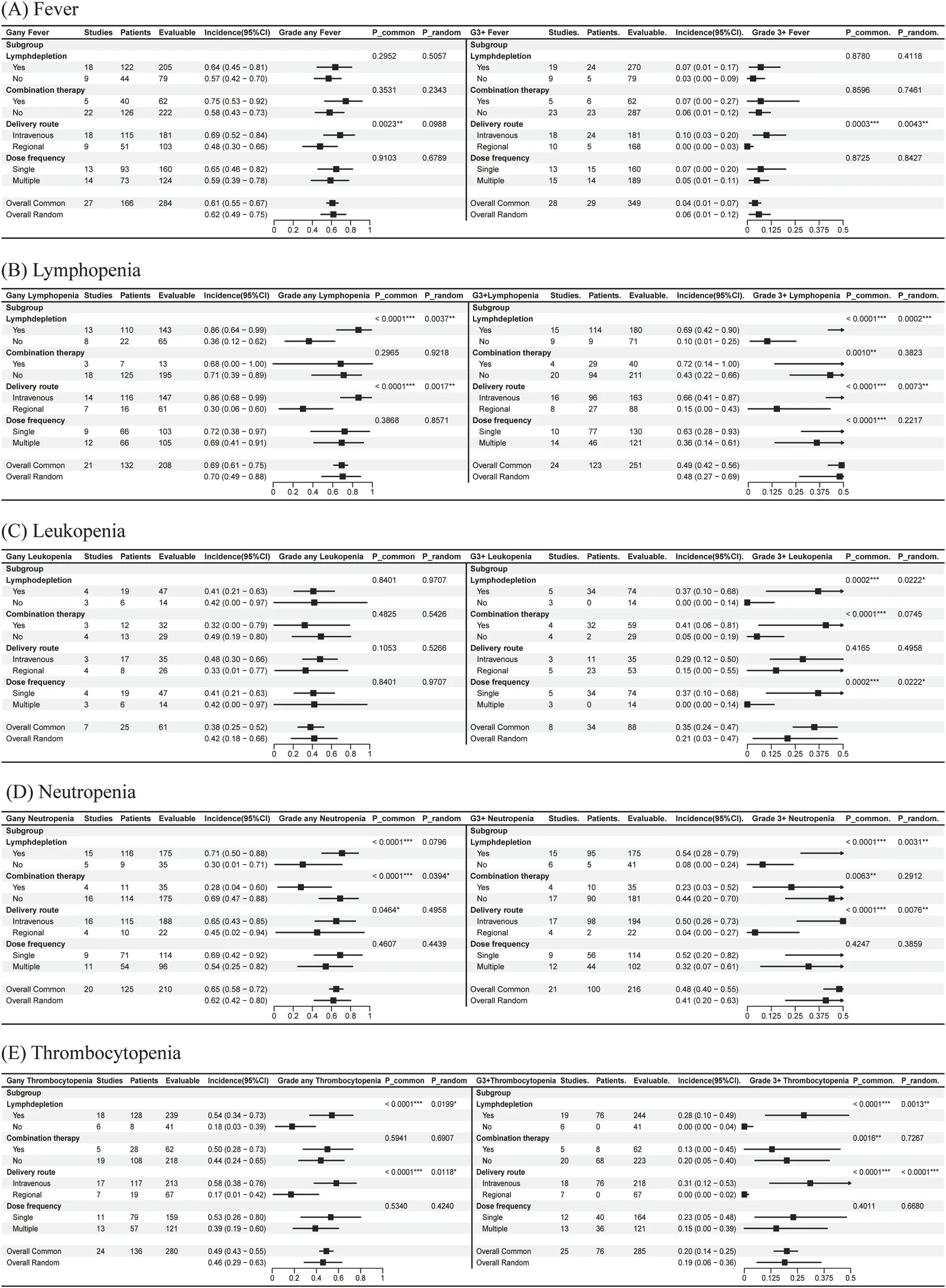
Forest plots for the incidence of any-grade adverse events (AEs) and grade ≥3 AEs for each adverse event: **(A)** Fever, **(B)** Lymphopenia, **(C)** Leukopenia, **(D)** Neutropenia, **(E)** Thrombocytopenia. Each forest plot was further stratified by lymphodepletion regimen, combination therapy, delivery route, and dose frequency. Squares in the plot indicate point estimates of the rates, and horizontal lines represent their respective 95% confidence intervals (95% CI). P_common: The P-value calculated by Common effects model(Fixed-effects model); P_random: The P-value calculated by the random-effects model.

Subgroup analyses were conducted to explore differences in AEs among CAR-T cell therapy recipients, focusing on the impact of lymphodepletion, combination therapy, administration route, and dose frequency (**Figure 2**, **Supplementary Figure S8**). Patients who underwent lymphodepletion exhibited significantly higher incidences of severe hematological toxicities than non-lymphodepletion counterparts. Specifically, rates of grade≥3 leukopenia (37% vs 0%, p = 0.0222), neutropenia (54% vs 8%, p < 0.0001), and anemia (65% vs 30%, p = 0.0349) were markedly elevated in the lymphodepletion group. Thrombocytopenia and lymphopenia followed similar trends: grade-any thrombocytopenia was 28% vs 10% (p < 0.0001), with grade≥3 thrombocytopenia at 54% vs 18% (p = 0.0199); any-grade lymphopenia reached 86% vs 36% (p = 0.0037), including grade≥3 lymphopenia at 28% vs 0% (p < 0.0001). Additionally, both grade-any neurotoxicity (75% vs 10%, p < 0.0001) and grade≥3 neurotoxicity (11% vs 0%, p < 0.0001) were significantly more prevalent in the central nervous system (CNS) patient subgroup compared with non-CNS counterparts (55). Intravenous infusion was associated with higher incidence of fever compared with regional delivery, including any-grade fever (69% vs 48%,p = 0.0023) and grade≥3 fever (10% vs 0%, p = 0.0003). The incidence of grade-any CRS was markedly higher in patients receiving intravenous administration (54%) compared with those treated via local administration (38%). Nevertheless, the rates of grade ≥3 CRS were relatively low in both groups, at 2% and 0%, respectively. Patients who received lymphodepletion had a higher incidence of grade-any CRS relative to those without lymphodepletion (55% versus 34%). This difference may be attributable to the limited sample size, the result did not reach statistical significance (p = 0.08). Dose frequency subgroup analysis revealed no significant differences in most AEs, except for grade≥3 leukopenia, which was more frequent in single dose group (37% vs 0%, p = 0.043). However, incidences of respiratory symptoms, gastrointestinal AEs (nausea, vomiting), and cutaneous AEs were comparable across all subgroups, with no statistically significant differences observed (**Supplementary Figure S8**).

### 3.4 Therapeutic efficacy

Across 52 studies with 712 evaluable patients (**Table 1**), complete response (CR) was observed in 19(2.7%) patients, partial response (PR) in 75 (10.5%), and stable disease (SD) in 335 patients. ORR and DCR varied substantially across studies (range:0-63%, 0-100%, respectively). Heterogeneity tests suggested low heterogeneity among studies for ORR (I² = 21%, p = 0.11) and DCR (I² = 24%, p = 0.07), according to common-effect model, the pooled ORR and DCR were 13% (95% CI: 11% -16%) and 63% (95% CI: 59% -67%), respectively (**Supplementary Figures S1**–**S4**).

Subgroup analyses revealed that ORR differed across the tumor types (**Supplementary Figure S2**), which was highest in neuroblastoma (34%) (15, 18, 23, 32, 35), followed by sarcoma(31%) (37, 56), colorectal cancer (CRC)(19%) (21), pancreatic cancer(16%) (20, 29, 64) and lowest in breast cancer(0%) (30, 44). However, for DCR (**Supplementary Figure S4**), Non-Small Cell Lung Cancer (NSCLC) and pancreatic cancer has the highest rate(70%) (20, 29, 33, 59, 64), followed by colorectal cancer(68%) (27, 40, 65), and while liver metastasis demonstrates the poorest outcomes(25%) (51, 52). DCR was significantly higher in patients with lymphodepletion(65%, 95%CI: 61-69%) than in those without lymphodepletion (54%, 95%CI: 47-61%, p = 0.0083, **Figure 3**). Similarly, ORR was markedly elevated in the lymphodepletion group (17%, 95%CI: 14-20%) compared with no lymphodepletion group (6%, 95%CI: 4-10%, p = 0.0002). In terms of CAR generation, ORR varied across CAR-T generations, with the highest numerical estimate observed for fourth-generation CAR-T cells (36%; 95%CI: 16-62%; two studies, n = 14). No significant difference in DCR was observed across generations.

**Figure 3:**
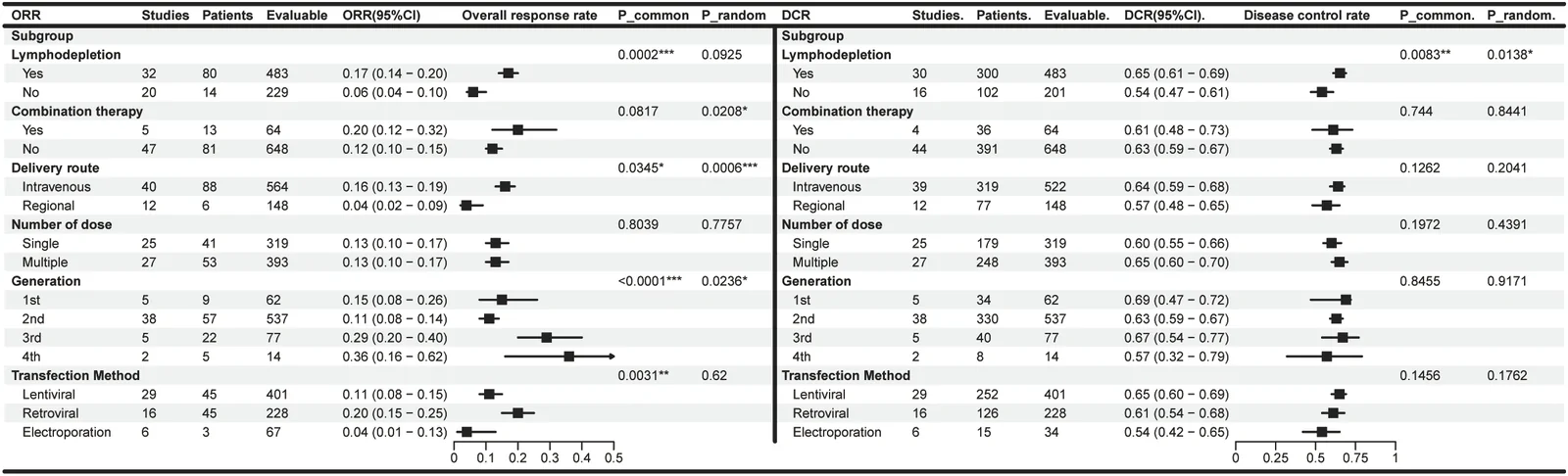
Forest plots of objective response rate (ORR) and disease control rate (DCR) stratified by subgroups of lymphodepletion regimen, combination therapy, delivery route, dose frequency, CAR-T generation, and transfection method. Squares in the plot indicate point estimates of the rates, and horizontal lines represent their respective 95% confidence intervals (95% CI). P_common: The P-value calculated by Common effects model(Fixed-effects model); P_random: The P-value calculated by the random-effects model.

ORR was significantly higher in patients receiving intravenous infusion (16%, 95%CI: 13-19%) than those with regional infusion (4%, 95%CI: 2-9%, p = 0.0345). Although DCR trended higher in the intravenous group (64% vs. 57%), this difference was not statistically significant (p = 0.1262). No significant differences were observed in pooled ORR or DCR between single-dose vs. multiple-dose subgroups, or between combination vs. monotherapy groups.

### 3.5 Survival outcomes

Sixteen studies involving 193 patients with available survival data were included in the survival analysis. The median OS was 10 months (95%CI: 7.50-12.50), and the median PFS was 2.48 months (95%CI: 2.00-2.83). Patients who achieved an overall response had a significantly longer median OS (20.5 months; 95%CI: 17.6-NA) than non-responders (8.2 months; 95%CI: 6.9-11.3; hazard ratio [HR]=0.3098, 95%CI: 0.1425-0.6734, p = 0.0018, **Supplementary Figure S6B**). Similarly, responders had a significantly longer median PFS (8.1 months; 95%CI: 4.0-19.6) than non-responders (2.0 months; 95%CI: 1.7-2.5; HR = 0.3111, 95%CI: 0.1966-0.4922, p < 0.0001, **Supplementary Figure S7B**). Among non-responders, patients with SD had a substantially improved median OS (14.1 months; 95%CI: 11.5-20.03) compared with those with PD (5.9 months; 95%CI: 4.4-7.9; HR = 0.4702, 95%CI: 0.2852-0.7753, p = 0.0026, **Supplementary Figure S6A**). Similarly, median PFS was markedly prolonged in SD patients (3.400 months; 95%CI: 2.866-4.400) versus PD patients (1.133 months; 95%CI: 1.00-1.27; HR = 0.2032, 95%CI: 0.1434-0.2878, p < 0.0001, **Supplementary Figure S7A**). However, subgroup analyses revealed no significant differences in OS or PFS between lymphodepletion and non-lymphodepletion, single-dose and multiple-dose, or combination-therapy and monotherapy groups.

### 3.6 Quality assessment

No evidence of funnel-plot asymmetry was detected for ORR (Egger’s test, P = 0.86; Begg’s test, P = 0.45), whereas significant asymmetry was observed for DCR (Egger’s test, P = 0.02; Begg’s test, P < 0.01), suggesting possible small-study effects or publication bias (**Supplementary Figure S5**).

### 3.7 Sensitivity analyses

Sensitivity analyses were conducted using alternative proportion-transformation methods. The pooled ORR and DCR estimates remained broadly consistent with those of the primary analysis (**Supplementary Table S4A**). Analysis of the collected individual participant data using the Mantel-Haenszel chi-squared test, stratified by treatment group, further supported the associations of lymphodepletion and administration route with therapeutic response (**Supplementary Table S4B**).

## 4 Discussion

To the best of our knowledge, this meta-analysis incorporates the most recent studies to systematically evaluate the safety and efficacy of CAR-T cells for solid tumors. Most of the studies adopted the Common Terminology Criteria for Adverse Events (CTCAE) to report adverse reactions, while some studies used the Lee method to report CRS (66, 67). Two major mechanisms contributing to AE in CAR-T therapy, one is on-target/on-tumor toxicity, which means activated by antigens expressed on tumor cells, CAR-T cells release a cascade of inflammatory cytokines (68), leading to clinical manifestations of CRS, which include pyrexia, rash, hypotension, even respiratory failure (67). Interleukin-6 (IL-6), secreted by immune cells, plays a pivotal role in CRS pathogenesis, thus severity of CRS can be mitigated by the IL-6 receptor inhibitor tocilizumab, particularly when used in conjunction with corticosteroids in clinical practice (69). We found that any-grade CRS was more frequent in the intravenous and lymphodepletion subgroup. However, due to the extremely low incidence of Grade3+ CRS (1%), no between-group differences were observed. Both any-grade and grade≥3 fever were significantly more frequent in patients who received intravenous delivery compared with those who received regional delivery, which suggests that local delivery improves the safety profile. Hematological toxicities dominated the top five adverse events, furthermore, our results revealed that patients who underwent lymphodepletion had significantly higher incidences of anemia, leukopenia, thrombocytopenia, neutropenia, and lymphopenia, compared with patients without lymphodepletion. These findings align with the known myelosuppressive effects of lymphodepleting chemotherapy regimens (70). Given the high incidence of hematologic toxicities, particularly overall and grade ≥3 lymphopenia, standardized hematologic monitoring across institutions would facilitate early risk identification and timely clinical management. The consistent application of monitoring schedules and toxicity-grading criteria would also improve the completeness and comparability of adverse-event reporting, thereby strengthening the evidence base for future clinical research.

Another is on-target/off-tumor toxicity originating from CAR-T cells targeting antigen expressed on normal cells, common manifestation of Immune effector cell-associated neurotoxicity syndrome (ICANS) including impaired cognitive dysfunction, headache, seizures (71). We found that neurological toxicity was exclusively associated with tumor type. Patients with CNS tumors demonstrated significantly higher incidences of both overall neurological toxicity and severe neurological toxicity following CAR-T cell infusion compared with patients with other tumor types. Additionally, as half of the studies employed intracranial or intraventricular administration, this approach exacerbated the incidence of neurological toxicity. This may reflect the potential vulnerability of pre-existing CNS disease to CAR-T cell-mediated neuroinflammation, therefore, a comprehensive neurological assessment of patients is essential before treatment. Clearly and proactively informing patients of the clinical manifestations and corresponding management strategies for adverse events can alleviate their psychological distress and enhance their confidence in treatment (72).

ORR varied substantially across different solid malignancies, with the highest rate observed in neuroblastoma (34%), followed by sarcoma (31%), whereas breast cancer showed the lowest rate (0%). This finding is consistent with that of Hou et al. (10), who reported the highest efficacy of CAR-T-cell therapy in neuroblastoma, with an ORR of 33%. Their meta-analysis, which included 22 studies involving 262 patients, reported an overall remission rate of 9% (95% CI: 4%-16%), comparable to the pooled ORR of 8% (95% CI: 5%-13%) estimated using the random-effects model in our meta-analysis. In our study, DCR was highest in NSCLC, followed by pancreatic and colorectal cancers, whereas liver metastases showed the poorest outcomes. This finding may be partly explained by the greater therapeutic resistance of distant metastases compared with primary tumors (57). Sensitivity analyses using alternative proportion-transformation methods and the available individual participant data yielded broadly consistent pooled ORR and DCR estimates and supported the associations of lymphodepletion and delivery route with therapeutic response. Despite subgroup analyses of several treatment-related factors, residual heterogeneity remained, which may partly reflect the inclusion of biologically diverse solid malignancies with substantial differences in tumor biology, antigen expression, and tumor microenvironment. Differences in disease status and baseline prognosis may also have contributed to the observed variability, as most included patients had advanced, metastatic, recurrent, or refractory disease. These potential sources of heterogeneity warrant further investigation.

The primary objectives of lymphodepletion are to deplete endogenous lymphocytes, thereby creating a favorable space for CAR-T cell expansion, and modulate the immune-suppressive microenvironment to enhance CAR-T cell persistence (73). Our findings indicate that lymphodepletion prior to infusion is associated with superior ORR and DCR compared to patients without lymphodepletion. Similar results were also identified in studies targeting hematologic malignancies and previous meta-analysis (9, 74).

Local administration of CAR-T cells promotes tumor infiltration and trafficking, eliciting robust antitumor immunity in preclinical models, thus proving safe and feasible for clinical translation (75). However, we also found that compared to local administration, intravenous administration has higher ORR as well as DCR, which is controversial to the conventional concept that local delivery can increase the concentration of cells and overcome the physical barrier posed by the TME (76). Modern oncology practice places greater emphasis on systemic effects for whole-body tumor control and control of detected or occult metastatic lesions. Intravenous administration, by targeting metastatic lesions and synergistically activating the systemic immune network, has emerged as the preferred approach for most antitumor agents. These results suggest that intravenous administration may represent the preferred delivery route to optimize therapeutic efficacy. However, more rigorous clinical investigations are warranted to validate this finding.

These findings support an individualized assessment of the efficacy-safety trade-off. Lymphodepletion and intravenous delivery may be considered for patients with rapidly progressive, refractory, or disseminated disease who have adequate performance status and hematologic reserve, whereas lymphodepletion should be used cautiously in patients at high risk of hematologic toxicity, and regional delivery may be preferable for localized, accessible tumors or patients unlikely to tolerate systemic toxicity. Given the predominantly early-phase, non-comparative evidence, these considerations remain hypothesis-generating and require confirmation in prospective comparative studies.

Several included studies evaluated emerging platforms, including bivalent EGFR/IL13Rα2 CAR-T cells and functionally enhanced constructs such as TGF-β-insensitive, IL-7 receptor-augmented, and RUNX3-expressing CAR-T cells. However, the limited number and small sample sizes of these studies precluded separate subgroup analyses. Although fourth-generation CAR-T cells showed a numerically higher ORR, this estimate was derived from only two studies involving 14 patients and should not be interpreted as evidence of a stepwise improvement across CAR-T generations. Similarly, the evidence for combination therapy was insufficient to reliably determine its effects on efficacy and adverse events, while substantial variation in infused cell doses and administration schedules limited the corresponding subgroup analyses.

Based on the available individual-level survival data, treatment response was associated with longer OS and PFS. Although lymphodepletion was associated with higher ORR and DCR in the overall analysis, no significant differences in OS or PFS were observed according to lymphodepletion status in the survival subset. Response is an early efficacy endpoint, whereas survival is also influenced by tumor type, baseline prognosis, and subsequent therapies. Lymphodepletion-related hematologic toxicity could attenuate longer-term benefits, but treatment-related mortality and causes of death were insufficiently reported to evaluate this possibility. Notably, survival data were available for only 193 of 712 patients (27.1%) from 16 studies. Whether the missingness was random could not be formally assessed and may have been systematic, as data availability depended on study-level reporting practices and follow-up completeness. The limited dataset also made within-tumor *post-hoc* comparisons underpowered and unstable. Therefore, these survival findings should be considered exploratory and interpreted cautiously.

MINORS scale was employed to assess the methodological quality of the included studies. The fifth item, ‘Unbiased assessment of the outcomes,’ which means blinded evaluation of outcome measures, but the vast majority of studies assessed outcomes without blinding. The eighth item, ‘Prospective calculation of the study size,’ was also considered, yet the majority of studies did not conduct sample size estimations, primarily due to the nature as Phase I clinical trials without control groups. This resulted in the majority of studies scoring zero on the 5th and 8th item. Encouragingly, the majority of studies scored relatively high on other items, so the quality is relative acceptable. Funnel-plot asymmetry and significant results from Egger’s and Begg’s tests for DCR indicated possible publication bias. Unlike comparative meta-analyses, this meta-analysis of proportions aimed to estimate event frequency rather than between-group treatment effects; nevertheless, selective publication may still distort the magnitude and precision of the pooled estimate. Moreover, because most included studies were small, single-arm phase I trials involving diverse tumor types and treatment strategies, small-study effects and genuine clinical heterogeneity may also have contributed to the asymmetry. Therefore, the pooled DCR estimate should be interpreted cautiously (77).

This study has several strengths. We integrated the most recent research on CAR-T cell therapies across various tumor types, thereby substantially increasing the sample size to systematically explore the ORR and DCR, as well as AE incidences at different grade. Despite variations in dosage levels, the number of administered doses, and cancer types within individual clinical trials, we endeavored to collect as much individual-level data as possible. This approach enabled more accurate subgroup analyses by minimizing potential intragroup heterogeneity, thereby enhancing the reliability and robustness of our results. Almost all identified studies were phase I single-arm trials. These trials are inherently characterized by small sample sizes and the absence of a control group. Additionally, adverse events were often incompletely or unclearly reported. These factors collectively complicate statistical analyses and may potentially compromise the validity of our findings. Moreover, most participants had advanced, metastatic, recurrent, or refractory tumors, limiting the generalizability of our findings to patients with earlier-stage disease. Such patients may have lower tumor burden, better performance status and immune reserve, and less treatment-related impairment, potentially resulting in different therapeutic responses and toxicity profiles. The benefit-risk balance may also differ because effective standard treatments are more commonly available in earlier-stage settings.

In conclusion, this study demonstrated that lymphodepletion and intravenous infusion were associated with higher ORR and DCR, but also with increased incidences of AEs. The balance between the therapeutic benefits and potential toxicity of CAR-T therapy remains an area for further investigation. There is an urgent need for more basic research to enhance the functions of CAR-T cells, and clinical trials are warranted to further elucidate the risks and benefits associated with various CAR-T cell strategies.

## Data Availability

The original contributions presented in the study are included in the article/**Supplementary Material**. Further inquiries can be directed to the corresponding authors.
