# Supplementary material for: The safety and efficacy of chimeric antigen receptor-T cell therapy in solid malignancies: a systematic review and meta-analysis
Source: Front Immunol. 2026 Sep 24;17:1957437. doi: 10.3389/fimmu.2026.1957437 (PMC13646177; doi:10.3389/fimmu.2026.1957437)

Supplementary Materials

The safety and efficacy of chimeric antigen receptor-T cell therapy in solid malignancies: a systematic review and meta-analysis

Table of Contents

Table S1. Search strategy: Full search terms for 4 databases

Table S2. Risk of bias assessments (methodological quality) by MINORS scale

Table S3. PRISMA 2020 Checklist

Table S4. Sensitive analysis of pooled ORR and DCR by (A) changing the methods of rate transformation and (B) individual participant data collected

Figure S1. Forest plots of individual study ORR estimates with an overall estimate

Figure S2. Forest plots of ORR by tumor type

Figure S3. Forest plots of individual study DCR estimates with an overall estimate

Figure S4. Forest plots of DCR by tumor type

Figure S5. Funnel plots to assess publication bias of studies investigating (A) ORR and (B) DCR (confirmed by Egger and Begg test)

Figure S6. Overall Survival Curves of the (A) response vs. no-response group, (B) stable disease vs. progressive disease group

Figure S7. Progression-Free Survival Curves of (A) response vs. no-response group, (B) stable disease vs. p rogressive disease group

Figure S8. Forest plots of incidence of any-grade AEs and grade 3+ AEs with subgroups, (A) Anemia, (B) Neurotoxicity, (C) Respiratory, (D) Nausea, (E) Vomit, (F) Cutaneous reaction

Table S1. Search strategy:Full search terms for 4 databases

	**Pubmed**
#1	(((Neoplasms [tiab] OR Neoplasm [tiab] OR Tumor [tiab] OR Tumors [tiab] OR Neoplasia [tiab] OR Neoplasias [tiab] OR Cancer [tiab] OR Cancers [tiab] OR Carcinoma [tiab] OR Carcinomas [tiab] OR Sarcoma [tiab] OR Sarcomas [tiab] OR Malignancy [tiab] OR Malignancies [tiab] OR Glioblastoma [tiab] OR Glioblastomas [tiab] OR Chromophobe [tiab] OR Glioma [tiab] OR Gliomas [tiab] OR Osteosarcoma [tiab] OR Osteosarcomas [tiab] OR Neuroglioma [tiab] OR Neuroblastoma* [tiab] OR Liposarcoma* [tiab] OR Fibrosarcoma* [tiab] OR Chondrosarcoma* [tiab] OR Adenocarcinoma* [tiab] OR Adenoma* [tiab] OR Cholangiocarcinoma* [tiab] OR Pheochromocytoma* [tiab] OR Paraganglioma* [tiab] OR Thymoma* [tiab] OR Astrocytoma* [tiab] OR Choriocarcinoma* [tiab] OR Fibrosarcoma* [tiab] OR Teratoma* [tiab] OR Mesothelioma* [tiab] OR Melanoma* [tiab] OR Carcinosarcoma*))
#2	((((((((((Immunotherapy, Adoptive[MeSH Terms]) OR (Chimeric Antigen Receptor Therapy[Title/Abstract])) OR (CAR T-Cell Therapy[Title/Abstract])) OR (CAR T Cell Therapy[Title/Abstract])) OR (CAR T-Cell Therapies[Title/Abstract])) OR (T-Cell Therapies, CAR[Title/Abstract])) OR (T-Cell Therapy, CAR[Title/Abstract])) OR (Therapies, CAR T-Cell[Title/Abstract])) OR (Therapy, CAR T-Cell[Title/Abstract])) OR ((((((((((((((((((Receptors, Chimeric Antigen[MeSH Terms]) OR (Antigen Receptors, Chimeric[Title/Abstract])) OR (Chimeric Antigen Receptors[Title/Abstract])) OR (Chimeric Antigen Receptor[Title/Abstract])) OR (Antigen Receptor, Chimeric[Title/Abstract])) OR (Receptor, Chimeric Antigen[Title/Abstract])) OR (Chimeric T-Cell Receptors[Title/Abstract])) OR (Chimeric T Cell Receptors[Title/Abstract])) OR (Receptors, Chimeric T-Cell[Title/Abstract])) OR (T-Cell Receptors, Chimeric[Title/Abstract])) OR (Artificial T-Cell Receptors[Title/Abstract])) OR (Artificial T Cell Receptors[Title/Abstract])) OR (Receptors, Artificial T-Cell[Title/Abstract])) OR (T-Cell Receptors, Artificial[Title/Abstract])) OR (Chimeric T-Cell Receptor[Title/Abstract])) OR (Chimeric T Cell Receptor[Title/Abstract])) OR (Receptor, Chimeric T-Cell[Title/Abstract])) OR (T-Cell Receptor, Chimeric[Title/Abstract])) OR (Chimeric Immunoreceptors[Title/Abstract])) OR (Immunoreceptors, Chimeric[Title/Abstract])))
#3	(((randomized controlled trial[pt] OR controlled clinical trial[pt] OR randomized[tiab] OR randomised[tiab] OR placebo[tiab] OR drug therapy[sh] OR randomly[tiab] OR trial[tiab] OR groups[tiab]) NOT (animals[mh] NOT humans[mh]))))
	#1 and #2 and #3
	**Embase**
#1	'neoplasms':ti,ab,kw OR 'neoplasm':ti,ab,kw OR 'tumor':ti,ab,kw OR 'tumors':ti,ab,kw OR 'neoplasia':ti,ab,kw OR 'neoplasias':ti,ab,kw OR 'cancer':ti,ab,kw OR 'cancers':ti,ab,kw OR 'carcinoma':ti,ab,kw OR 'carcinomas':ti,ab,kw OR 'sarcoma':ti,ab,kw OR 'sarcomas':ti,ab,kw OR 'malignancy':ti,ab,kw OR 'malignancies':ti,ab,kw OR 'glioblastoma':ti,ab,kw OR 'glioblastomas':ti,ab,kw OR 'chromophobe':ti,ab,kw OR 'glioma':ti,ab,kw OR 'gliomas':ti,ab,kw OR 'osteosarcoma':ti,ab,kw OR 'osteosarcomas':ti,ab,kw OR 'neuroglioma':ti,ab,kw OR 'neuroblastoma$':ti,ab,kw OR 'liposarcoma$':ti,ab,kw OR 'chondrosarcoma$':ti,ab,kw OR 'adenocarcinoma$':ti,ab,kw OR 'adenoma$':ti,ab,kw OR 'cholangiocarcinoma$':ti,ab,kw OR 'pheochromocytoma$ ':ti,ab,kw OR 'paraganglioma$':ti,ab,kw OR 'thymoma$':ti,ab,kw OR 'astrocytoma$':ti,ab,kw OR 'choriocarcinoma$':ti,ab,kw OR 'fibrosarcoma$':ti,ab,kw OR 'teratoma$':ti,ab,kw OR 'mesothelioma$':ti,ab,kw OR 'melanoma$':ti,ab,kw OR 'carcinosarcoma$':ti,ab,kw
#2	'chimeric antigen receptor'/syn OR 'chimeric antigen receptor' OR 'chimeric antigen receptor immunotherapy'/syn OR 'chimeric antigen receptor immunotherapy' OR 'chimeric antigen receptor t-cell'/syn OR 'chimeric antigen receptor t-cell' OR 'car t':ti,ab,kw
#3	'randomized controlled trial':ti,ab,kw OR 'controlled clinical trial':ti,ab,kw OR 'clinical trial':ti,ab,kw OR 'randomized clinical trial':ti,ab,kw OR 'rct':ti,ab,kw OR 'randomized':ti,ab,kw OR 'randomised':ti,ab,kw OR 'randomly':ti,ab,kw OR 'placebo':ti,ab,kw
	#1 and #2 and #3
	**Web of Science**
#1	TS=(Neoplasms OR Neoplasm OR Tumor OR Tumors OR Neoplasia OR Neoplasias OR Cancer OR Cancers OR Carcinoma OR Carcinomas OR Sarcoma OR Sarcomas OR Malignancy OR Malignancies OR Glioblastoma OR Glioblastomas OR Chromophobe OR Glioma OR Gliomas OR Osteosarcoma OR Osteosarcomas OR Neuroglioma OR Neuroblastoma$ OR Liposarcoma$ OR Fibrosarcoma$ OR Chondrosarcoma$ OR Adenocarcinoma$ OR Adenoma$ OR Cholangiocarcinoma$ OR Pheochromocytoma$ OR Paraganglioma$ OR Thymoma$ OR Astrocytoma$ OR Choriocarcinoma$ OR Fibrosarcoma$ OR Teratoma$ OR Mesothelioma$ OR Melanoma$ OR Carcinosarcoma$)
#2	TS=(Chimeric Antigen Receptor OR Artificial T-Cell Receptors OR Artificial T Cell Receptors OR Chimeric T-Cell Receptor OR Chimeric T Cell Receptor OR Chimeric Immunoreceptor OR CAR-T OR CAR)
#3	TS=(randomized controlled trial OR controlled clinical trial OR clinical trial OR randomized clinical trial OR rct OR randomized OR randomly OR placebo OR trial)
	#1 and #2 and #3
	**Cochrane Library**
#1	(Therapy, CAR T-Cell):ti,ab,kw OR (CAR T-Cell Therapies):ti,ab,kw OR (CAR T Cell Therapy):ti,ab,kw OR (CAR T-Cell Therapy):ti,ab,kw OR (Therapies, CAR T-Cell):ti,ab,kw OR (Therapy, CAR T-Cell):ti,ab,kw OR (CAR T-Cell Therapies):ti,ab,kw OR (CAR T Cell Therapy):ti,ab,kw OR (T-Cell Therapies, CAR):ti,ab,kw OR (CAR T-Cell Therapy):ti,ab,kw OR (Chimeric Antigen Receptor Therapy):ti,ab,kw OR (Therapies, CAR T-Cell):ti,ab,kw OR (T-Cell Therapy, CAR):ti,ab,kw OR (Cellular Immunotherapies, Adoptive):ti,ab,kw OR (Adoptive Cellular Immunotherapies):ti,ab,kw OR (Adoptive Cellular Immunotherapy):ti,ab,kw OR (Immunotherapies, Adoptive Cellular):ti,ab,kw OR (Cellular Immunotherapy, Adoptive):ti,ab,kw OR (Immunotherapy, Adoptive Cellular):ti,ab,kw OR (Adoptive Immunotherapies):ti,ab,kw OR (Adoptive Immunotherapy):ti,ab,kw OR (Immunotherapies, Adoptive):ti,ab,kw
#2	(neoplasms):ti,ab,kw OR (neoplasm):ti,ab,kw OR (tumor):ti,ab,kw OR (tumors):ti,ab,kw OR (neoplasia):ti,ab,kw OR (neoplasias):ti,ab,kw OR (cancer):ti,ab,kw OR (cancers):ti,ab,kw OR (carcinoma):ti,ab,kw OR (carcinomas):ti,ab,kw OR (sarcoma):ti,ab,kw OR (sarcomas):ti,ab,kw OR (malignancy):ti,ab,kw OR (malignancies):ti,ab,kw OR (glioblastoma):ti,ab,kw OR (glioblastomas):ti,ab,kw OR (chromophobe):ti,ab,kw OR (glioma):ti,ab,kw OR (gliomas):ti,ab,kw OR (osteosarcoma):ti,ab,kw OR (osteosarcomas):ti,ab,kw OR (neuroglioma):ti,ab,kw OR (neuroblastoma):ti,ab,kwOR(liposarcoma):ti,ab,kw OR (chondrosarcoma):ti,ab,kwOR(adenocarcinoma):ti,ab,kw OR (adenoma):ti,ab,kw OR (cholangiocarcinoma):ti,ab,kw OR (pheochromocytoma$):ti,ab,kw OR (paraganglioma):ti,ab,kw OR(thymoma):ti,ab,kw OR (astrocytoma):ti,ab,kwOR(choriocarcinoma):ti,ab,kw OR (fibrosarcoma):ti,ab,kw OR (teratoma):ti,ab,kw OR (mesothelioma):ti,ab,kwOR(melanoma):ti,ab,kw OR (carcinosarcoma$):ti,ab,kw
#3	(randomized controlled trial):ti,ab,kw OR (controlled clinical trial):ti,ab,kw OR (clinical trial):ti,ab,kw OR (randomized clinical trial):ti,ab,kw OR (RCT):ti,ab,kw OR (randomized):ti,ab,kw OR (randomised):ti,ab,kw OR (randomly):ti,ab,kw OR (placebo):ti,ab,kw
	#1 and #2 and #3

Table S2. Risk of bias assessments(methodological quality) by MINORS scale

Author(year)	A clearly stated aim	Inclusion of consecutive patients	Prospective collection of data	Endpoints appropriate to the aim of the study	Unbiased assessment of the study endpoint	Follow-up period appropriate to the aim of the study	Loss to follow up less than 5%	Prospective calculation of the study size	Score
Prasad S. Adusumilli(2021)	2	2	2	2	0	2	2	1	13
Donald M. O'Rourke(2017)	2	2	2	2	0	2	2	1	13
Gregory L. Beatty(2018)	2	0	2	2	0	0	0	0	6
Julie R Park(2007)	2	2	2	2	0	0	0	0	8
Yang Liu(2020)	2	2	2	2	0	2	2	0	12
Crystal U. Louis(2011)	2	2	2	2	0	2	2	0	12
Andras Heczey(2017)	2	2	2	2	0	2	2	0	12
Donghua Shi(2020)	2	2	2	2	0	2	2	0	12
Kaichao Feng(2016)	2	2	2	2	0	2	2	0	12
Mackensen.A(2023)	2	2	2	2	0	2	0	0	10
Hanren Dai(2020)	2	2	2	2	0	2	2	0	12
Dan Li(2023)	2	2	2	2	0	2	2	0	12
F. Del Bufalo(2023)	2	2	2	2	2	2	2	0	14
Lihua Yu(2022)	2	2	2	2	0	2	2	0	12
Nabil Ahmed(2017)	2	2	2	2	0	2	2	0	12
Steven C. Katz(2020)	2	2	2	2	0	2	2	0	12
Sophie Papa(2023)	2	2	2	2	0	2	2	0	12
Nicholas A. Vitanza(2023)	2	2	2	2	0	0	0	0	8
Yajun Zhang(2021)	2	2	2	2	0	1	0	0	9
Chengcheng Zhang(2017)	2	2	2	2	0	0	0	0	8
Steven C. Katz(2015)	2	1	2	2	1	2	2	0	12
Yelei Guo(2018)	2	2	2	2	0	2	2	0	12
Kaichao Feng(2017)	2	2	2	2	0	1	0	0	9
Andrew R. Haas(2019)	2	2	2	2	0	2	2	0	12
Richard P. Junghans(2016)	2	1	2	2	0	0	0	0	7
Payal D. Shah(2023)	2	2	2	2	0	2	2	0	12
Stephanie L. Goff(2019)	2	1	2	2	0	0	0	0	7
Vivek Narayan(2022)	2	2	2	2	0	2	2	0	12
Qihan Fu(2023)	2	2	2	2	0	2	2	0	12
Zhuohao Liu(2023)	2	2	2	2	0	2	2	0	12
Julia Tchou(2017)	2	2	2	2	0	0	0	0	8
Fiona C. Thistlethwaite(2017)	2	2	2	2	0	2	0	0	10
Changsong Qi(2024)	2	2	2	2	0	2	2	0	12
Sumanta K. Pal(2024)	2	1	2	2	0	0	0	0	7
Meenakshi Hegde(2024)	2	2	2	2	0	2	2	0	12
Christine E. Brown(2024)	2	2	2	2	0	2	2	0	12
Tessa Gargett(2024)	2	2	2	2	0	2	2	0	12
Frank Y. Lin, MD(2024)	2	2	2	2	0	2	2	0	12
Tanya B. Dorff(2024)	2	2	2	2	0	2	2	0	12
Naifei Chen(2024)	2	2	2	2	0	2	2	0	12
Tongpeng Xu(2024)	2	2	2	2	0	2	2	0	12
Stephen J. Bagley(2024)	2	2	2	2	2	2	2	0	14
Navin Pinto(2024)	2	2	2	2	0	2	2	0	12
Nicholas A Vitanza(2025)	2	2	2	2	0	2	2	0	12
Juliane Gust(2025)	2	2	2	2	0	2	2	0	12
Stein, M. N.(2024)	2	2	2	2	0	2	2	0	12
Michael E. Barish(2025)	2	2	2	2	0	2	2	0	12
Changsong Qi(2026)	2	2	2	2	0	2	2	0	12
Yongqiang Yang(2026)	2	2	2	2	0	2	2	0	12
Susan Slovin(2026)	2	2	2	2	0	2	2	0	12
Yang Gao(2026)	2	2	2	2	0	2	2	0	12
Stephen J. Bagley(2025)	2	2	2	2	0	2	2	0	12

The methodological quality of the studies were independently assessed using MINORS(methodological index for non-randomized studies) which contained 12 items,each item was scored from 0 to 2; 0 indicating that it was not reported, 1 indicating inadequate reporting, and 2 indicating that it was reported adequately

Table S3. PRISMA 2020 Checklist

PRISMA 2020 Checklist

Section and Topic	Item #	Checklist item	Location where item is reported
**TITLE**			
Title	1	Identify the report as a systematic review.	Page1
**ABSTRACT**			
Abstract	2	See the PRISMA 2020 for Abstracts checklist.	Page1
**INTRODUCTION**			
Rationale	3	Describe the rationale for the review in the context of existing knowledge.	Page1
Objectives	4	Provide an explicit statement of the objective(s) or question(s) the review addresses.	Page1
**METHODS**			
Eligibility criteria	5	Specify the inclusion and exclusion criteria for the review and how studies were grouped for the syntheses.	Page3
Information sources	6	Specify all databases, registers, websites, organisations, reference lists and other sources searched or consulted to identify studies. Specify the date when each source was last searched or consulted.	Page3
Search strategy	7	Present the full search strategies for all databases, registers and websites, including any filters and limits used.	Appendix p2
Selection process	8	Specify the methods used to decide whether a study met the inclusion criteria of the review, including how many reviewers screened each record and each report retrieved, whether they worked independently, and if applicable, details of automation tools used in the process.	Page3
Data collection process	9	Specify the methods used to collect data from reports, including how many reviewers collected data from each report, whether they worked independently, any processes for obtaining or confirming data from study investigators, and if applicable, details of automation tools used in the process.	Page3,8
Data items	10a	List and define all outcomes for which data were sought. Specify whether all results that were compatible with each outcome domain in each study were sought (e.g. for all measures, time points, analyses), and if not, the methods used to decide which results to collect.	Page3
	10b	List and define all other variables for which data were sought (e.g. participant and intervention characteristics, funding sources). Describe any assumptions made about any missing or unclear information.	Page3
Study risk of bias assessment	11	Specify the methods used to assess risk of bias in the included studies, including details of the tool(s) used, how many reviewers assessed each study and whether they worked independently, and if applicable, details of automation tools used in the process.	Page3,4,6 Appendix.p3
\Effect measures	12	Specify for each outcome the effect measure(s) (e.g. risk ratio, mean difference) used in the synthesis or presentation of results.	Page3
Synthesis methods	13a	Describe the processes used to decide which studies were eligible for each synthesis (e.g. tabulating the study intervention characteristics and comparing against the planned groups for each synthesis (item #5)).	Page3
	13b	Describe any methods required to prepare the data for presentation or synthesis, such as handling of missing summary statistics, or data conversions.	Page3
	13c	Describe any methods used to tabulate or visually display results of individual studies and syntheses.	Page3
	13d	Describe any methods used to synthesize results and provide a rationale for the choice(s). If meta-analysis was performed, describe the model(s), method(s) to identify the presence and extent of statistical heterogeneity, and software package(s) used.	Page3
	13e	Describe any methods used to explore possible causes of heterogeneity among study results (e.g. subgroup analysis, meta-regression).	Page3
	13f	Describe any sensitivity analyses conducted to assess robustness of the synthesized results.	Page3
Reporting bias assessment	14	Describe any methods used to assess risk of bias due to missing results in a synthesis (arising from reporting biases).	-
Certainty	15	Describe any methods used to assess certainty (or confidence) in the body of evidence for an outcome.	Page3

PRISMA 2020 Checklist

Section and Topic	Item #	Checklist item	Location where item is reported
assessment			
**RESULTS**			
Study selection	16a	Describe the results of the search and selection process, from the number of records identified in the search to the number of studies included in the review, ideally using a flow diagram.	Page4
	16b	Cite studies that might appear to meet the inclusion criteria, but which were excluded, and explain why they were excluded.	-
Study characteristics	17	Cite each included study and present its characteristics.	Page4
Risk of bias in studies	18	Present assessments of risk of bias for each included study.	Page4
Results of individual studies	19	For all outcomes, present, for each study: (a) summary statistics for each group (where appropriate) and (b) an effect estimate and its precision (e.g. confidence/credible interval), ideally using structured tables or plots.	Page4
Results of syntheses	20a	For each synthesis, briefly summarise the characteristics and risk of bias among contributing studies.	Page4
	20b	Present results of all statistical syntheses conducted. If meta-analysis was done, present for each the summary estimate and its precision (e.g. confidence/credible interval) and measures of statistical heterogeneity. If comparing groups, describe the direction of the effect.	Page4
	20c	Present results of all investigations of possible causes of heterogeneity among study results.	Page5
	20d	Present results of all sensitivity analyses conducted to assess the robustness of the synthesized results.	Page6
Reporting biases	21	Present assessments of risk of bias due to missing results (arising from reporting biases) for each synthesis assessed.	Appendix.p3
Certainty of evidence	22	Present assessments of certainty (or confidence) in the body of evidence for each outcome assessed.	Page6
**DISCUSSION**			
Discussion	23a	Provide a general interpretation of the results in the context of other evidence.	Page6
	23b	Discuss any limitations of the evidence included in the review.	Page8
	23c	Discuss any limitations of the review processes used.	Page8
	23d	Discuss implications of the results for practice, policy, and future research.	Page7,8
**OTHER INFORMATION**			
Registration and protocol	24a	Provide registration information for the review, including register name and registration number, or state that the review was not registered.	Page1,3
	24b	Indicate where the review protocol can be accessed, or state that a protocol was not prepared.	-
	24c	Describe and explain any amendments to information provided at registration or in the protocol.	-
Support	25	Describe sources of financial or non-financial support for the review, and the role of the funders or sponsors in the review.	Page8
Competing interests	26	Declare any competing interests of review authors.	Page8
Availability of data, code and other materials	27	Report which of the following are publicly available and where they can be found: template data collection forms; data extracted from included studies; data used for all analyses; analytic code; any other materials used in the review.	Page8

From: Page MJ, McKenzie JE, Bossuyt PM, Boutron I, Hoffmann TC, Mulrow CD, et al. The PRISMA 2020 statement: an updated guideline for reporting systematic reviews. BMJ 2021;372:n71. doi: 10.1136/bmj.n71

For more information, visit: http://www.prisma-statement.org/

Table S4. Sensitive analysis of pooled ORR and DCR by (A)changing the methods of rate transformation and (B)Individual participant data collected

(A)

	ORR(95CI%)	PRAW				DCR(95CI%)	PRAW	
Subgroup		P_random	P_common		Subgroup		P_random	P_common
Lymphodepletion		0.1452	0.0043		Lymphodepletion		0.0375	0.0015
Yes	0.14(0.08-0.20)				Yes	0.65(0.59-0.72)		
No	0.04(0.01-0.07)				No	0.49(0.35-0.63)		
Combination therapy		0.7679	0.477		Combination therapy		0.9636	0.4686
Yes	0.11(0.00-0.23)				Yes	0.59(0.47-0.71)		
No	0.09(0.05-0.13)				No	0.59(0.52-0.67)		
Delivery route		0.0379	0.4847		Delivery route		0.0386	0.0001
Intravenous	0.11(0.06-0.16)				Intravenous	0.64(0.58-0.71)		
Regional	0.05(0.01-0.08)				Regional	0.45(0.29-0.62)		
Dose frequency		0.6994	0.7202		Dose frequency		0.2725	0.2725
Single	0.10(0.05-0.15)				Single	0.63(0.55-0.71)		
Multiple	0.08(0.03-0.14)				Multiple	0.55(0.44-0.66)		
Generation		0.2792	0.2597		Generation		0.2464	0.1894
1st	0.13(0.00-0.27)				1st	0.54(0.30-0.78)		
2nd	0.05(0.03-0.07)				2nd	0.58(0.50-0.66)		
3rd	0.20(0.00-0.45)				3rd	0.65(0.47-0.82)		
4th	0.32(0.00-0.65)				4th	0.57(0.32-0.83)		
Transfection Method		0.2318	0.7318		Transfection Method		0.0693	0.0041
Lentiviral	0.05(0.02-0.08)				Lentiviral	0.61(0.51-0.71)		
Retroviral	0.15(0.06-0.24)				Retroviral	0.59(0.51-0.38)		
Electroporation	0.04(0.00-0.13)				Electroporation	0.40(0.16-0.65)		
Overall Common	0.06(0.04-0.08)				Overall Common	0.63(0.60-0.67)		
Overall Random	0.09(0.05-0.13)				Overall Random	0.59(0.52-0.66)		
I2:57% p<0.01 PRAW:Untransformed proportions					I2:72% p<0.01 PRAW:Untransformed proportions			

(B)The Mantel - Haenszel chi - squared test(Stratify according to the treatment groups) was employed to explore the associations between lymphodepletion, delivery route, and therapeutic benefits.

Lymphodepletion(LD)	Delivery	Response	No-Response	OR(95CI%)	MH χ2	Pvalue
LD	Intravenous	60	235	2.66(1.08-6.53)	4.319	0.038*
	Regional	3	19			
No-LD	Intravenous	13	96			
	Regional	3	96			

Lymphodepletion(LD)	Delivery	Controlled Disease	Progressive Disease	OR(95CI%)	MH χ2	Pvalue
LD	Intravenous	211	84	1.30(0.81-2.08)	0.939	0.333
	Regional	15	7			
No-LD	Intravenous	62	47			
	Regional	49	50			

Delivery	Lymphodepletion(LD)	Response	No-Response	OR(95CI%)	MH χ2	Pvalue
Intravenous	LD	60	235	2.07(1.12-3.81)	5.185	0.023*
	No-LD	13	96			
Regional	LD	3	19			
	No-LD	3	96			

Delivery	Lymphodepletion(LD)	Controlled Disease	Progressive Disease	OR(95CI%)	MH χ2	Pvalue
Intravenous	LD	211	84	1.96(1.29-2.96)	9.580	0.002**
	No-LD	62	47			
Regional	LD	15	7			
	No-LD	49	50			

Figure S1. Forest plots of individual study ORR estimates with an overall estimate

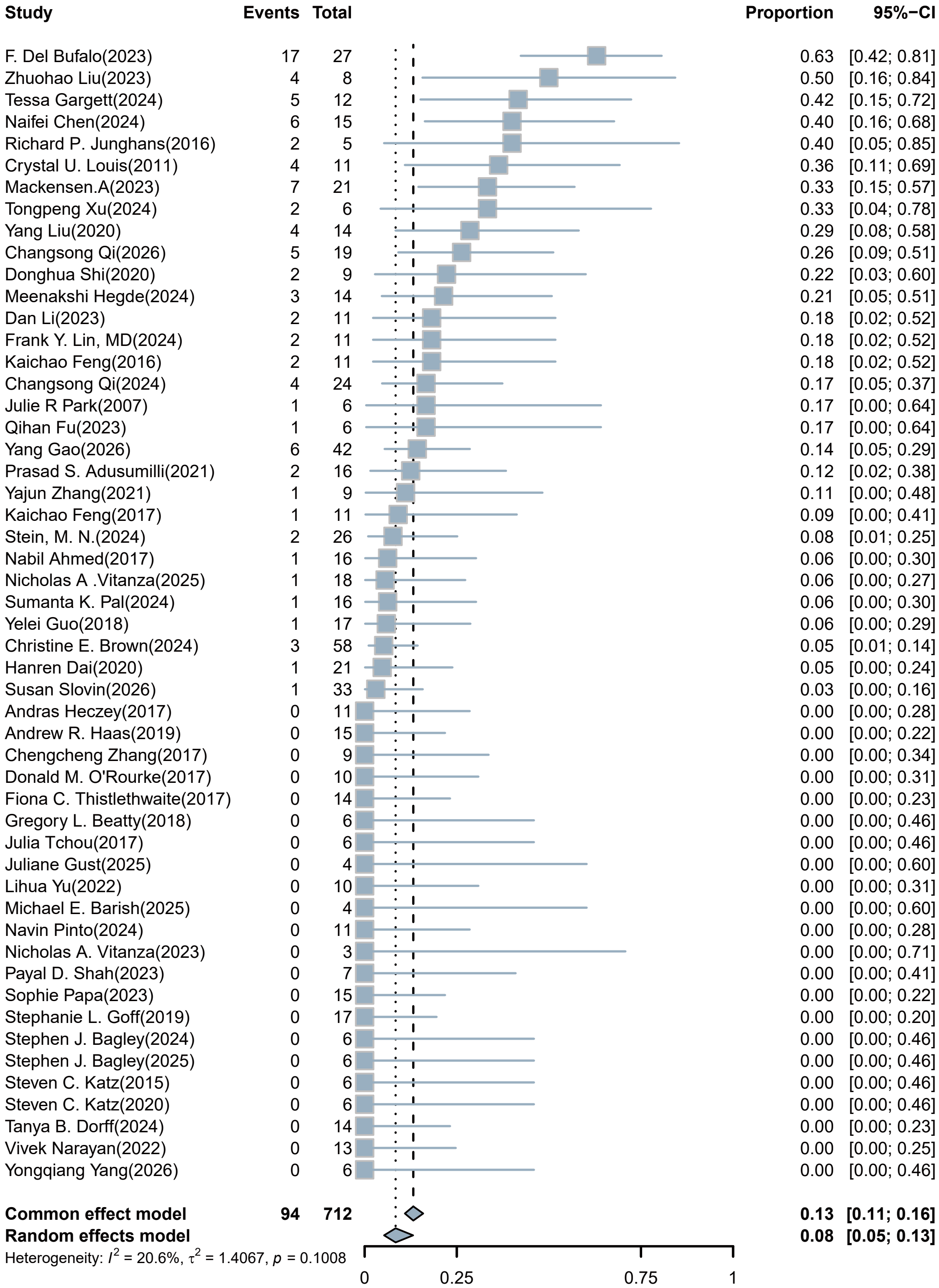

Figure S2. Forest plots of ORR by tumor type

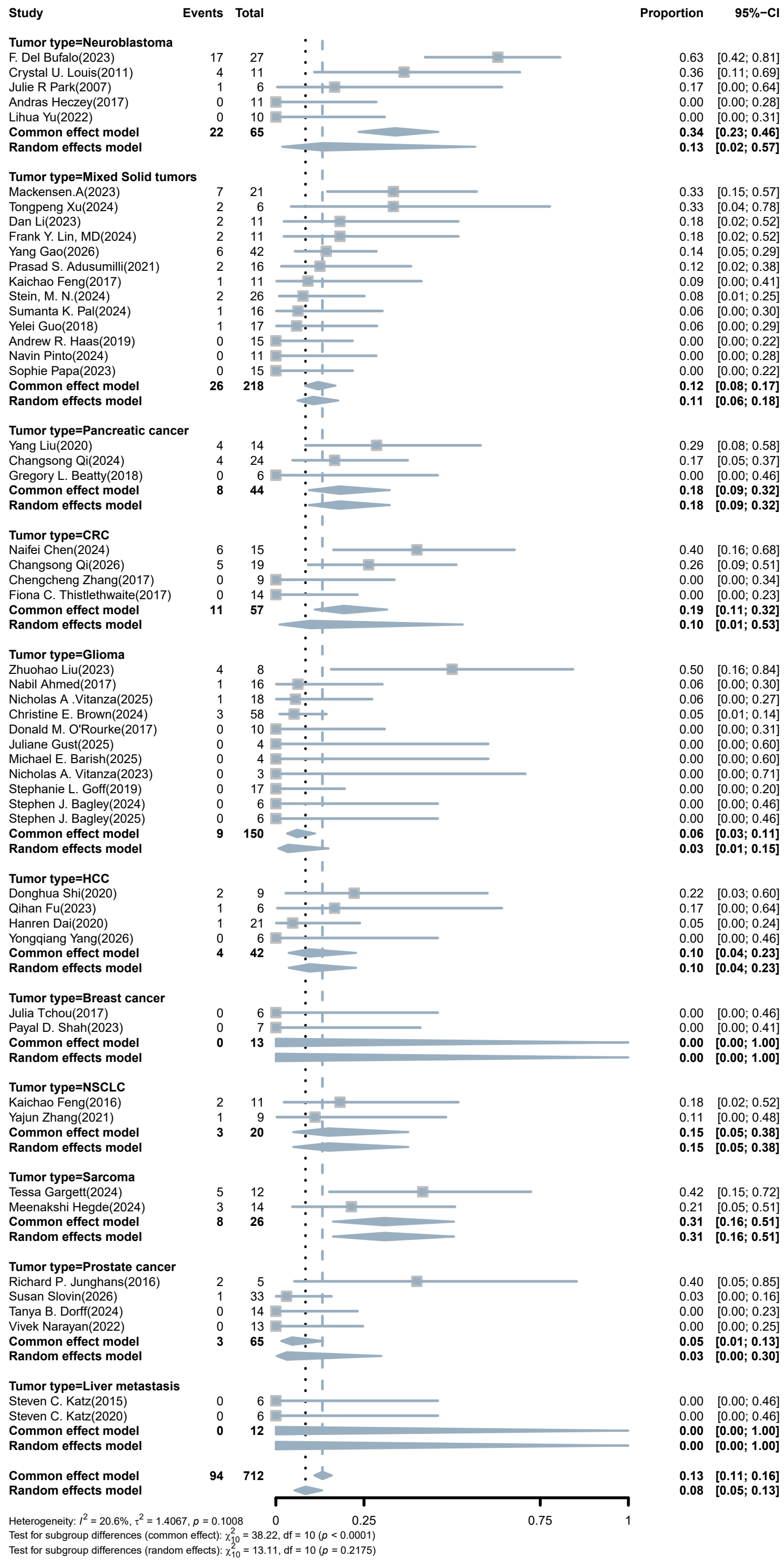

Figure S3. Forest plots of individual study DCR estimates with an overall estimate

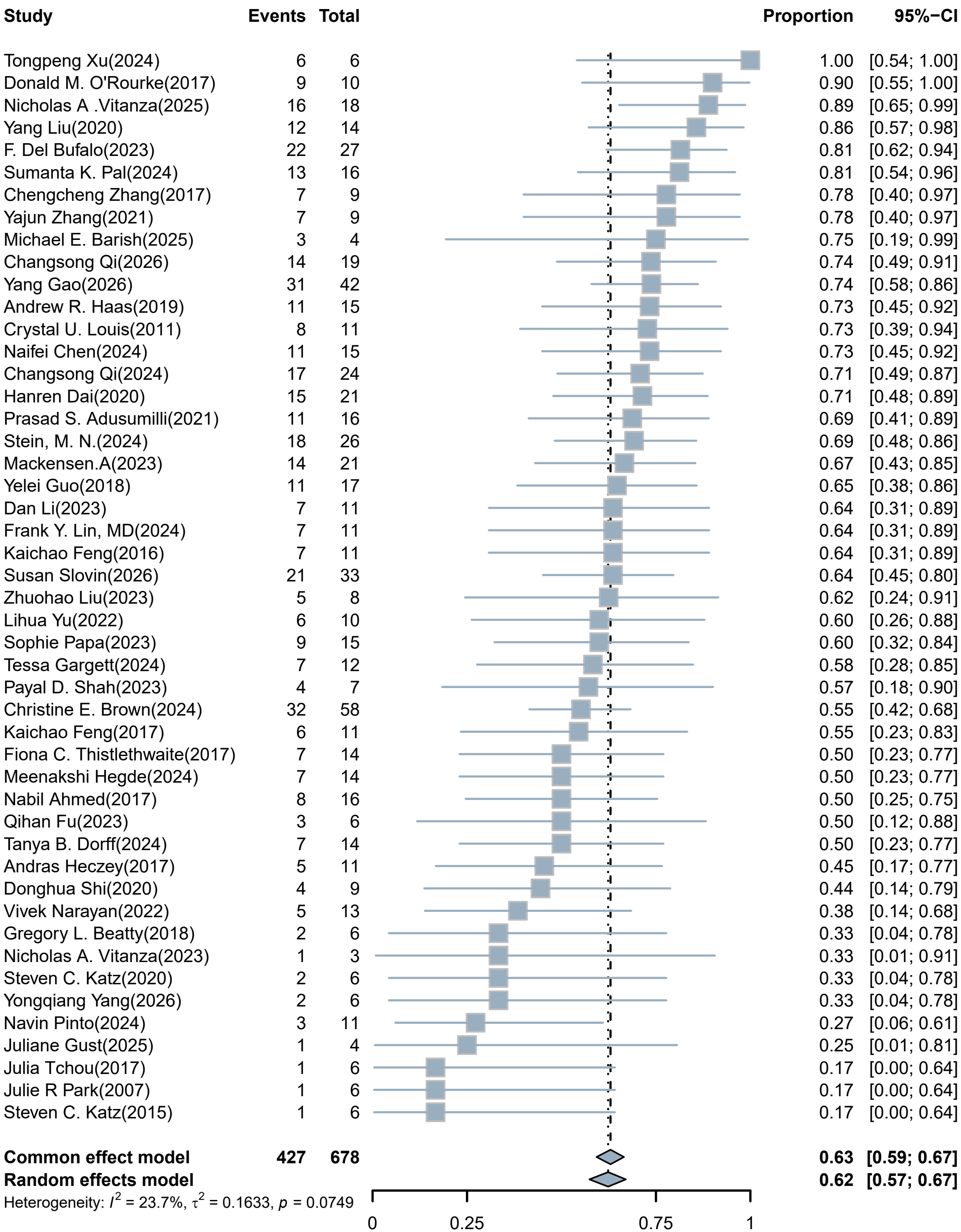

Figure S4. Forest plots of DCR by tumor type

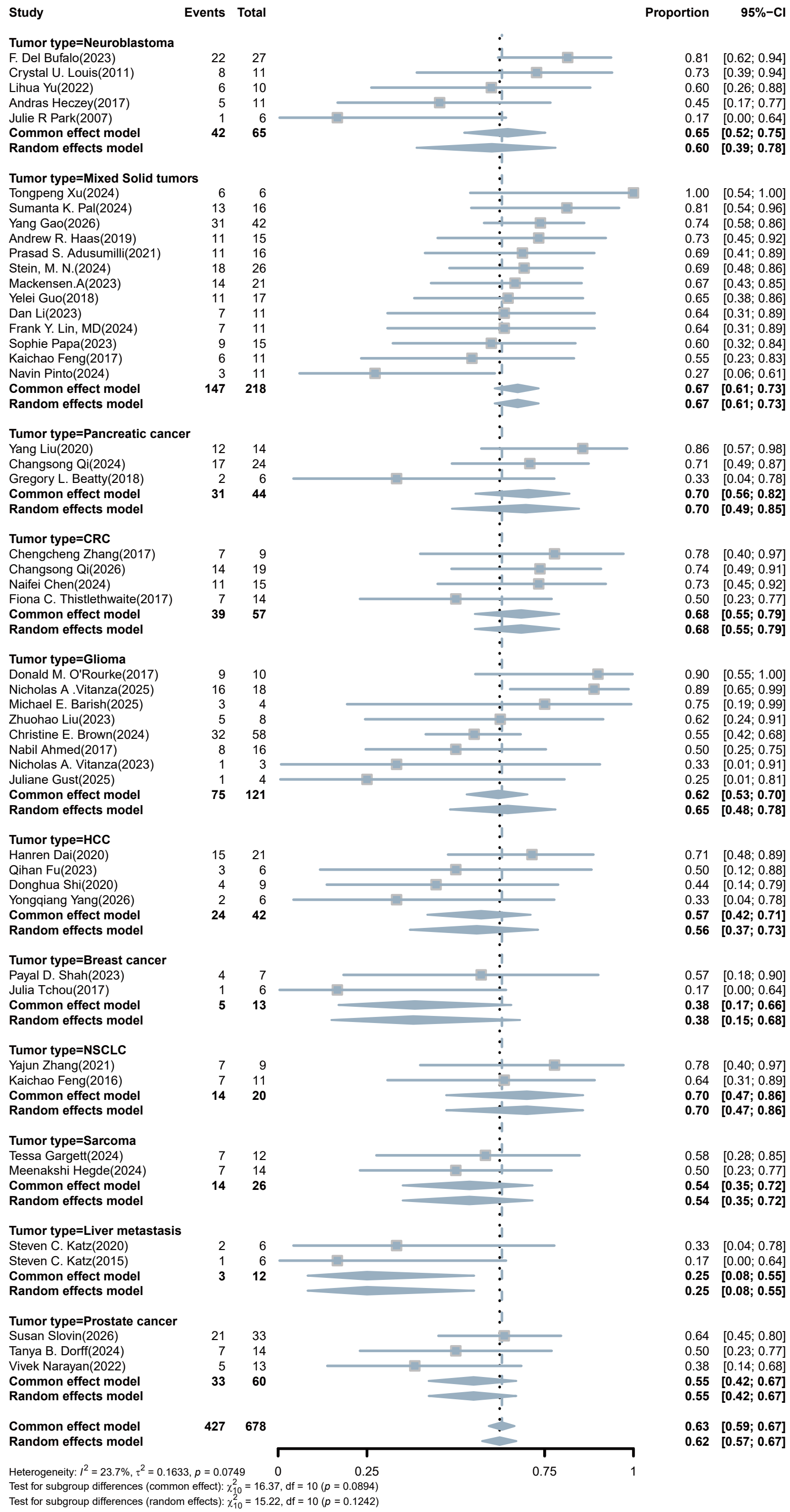

Figure S5. Funnel plots to assess publication bias of studies investigating (A) ORR and (B) DCR (comfirmed by Egger and Begg. test)

(A)

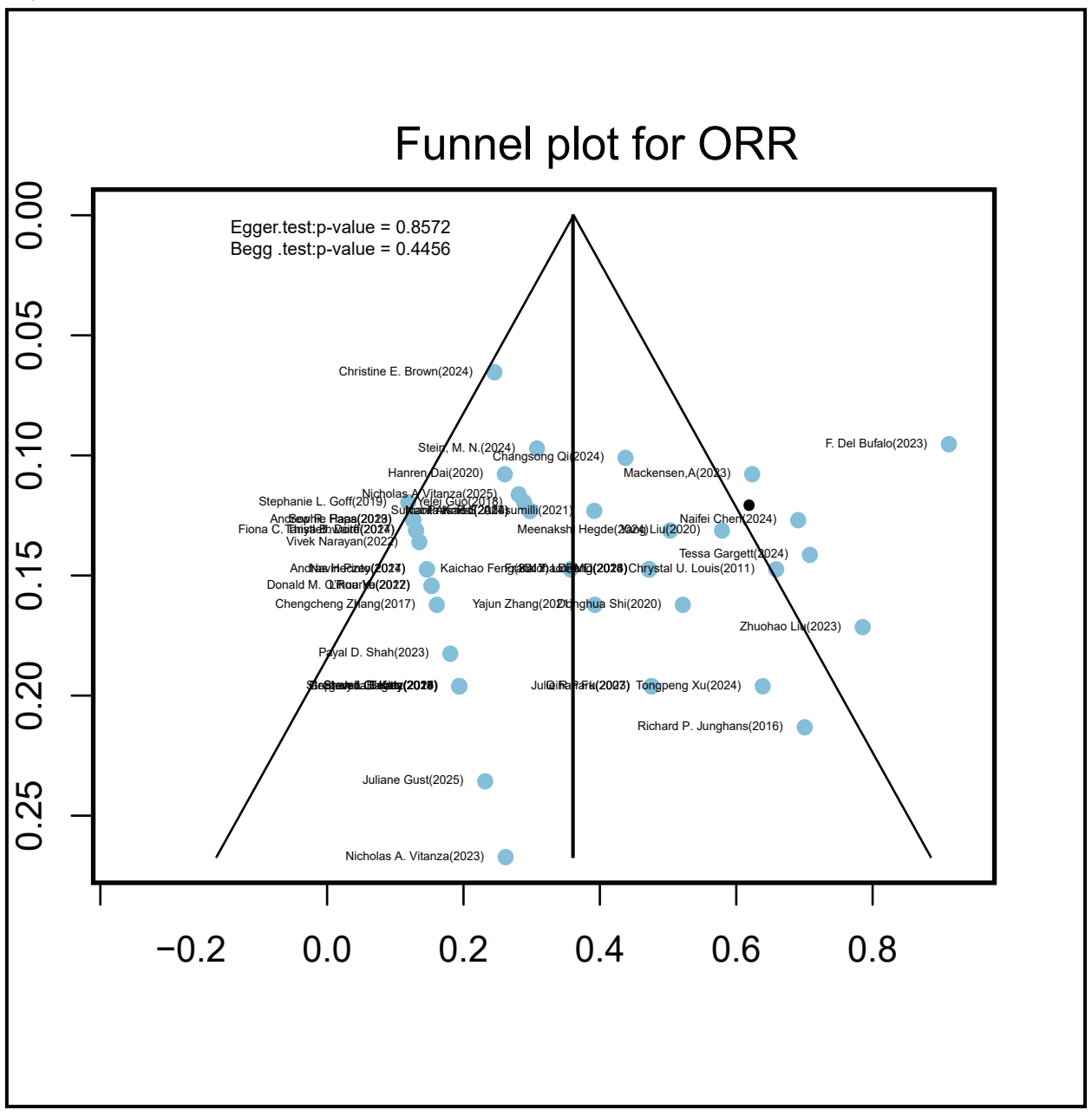

(B)

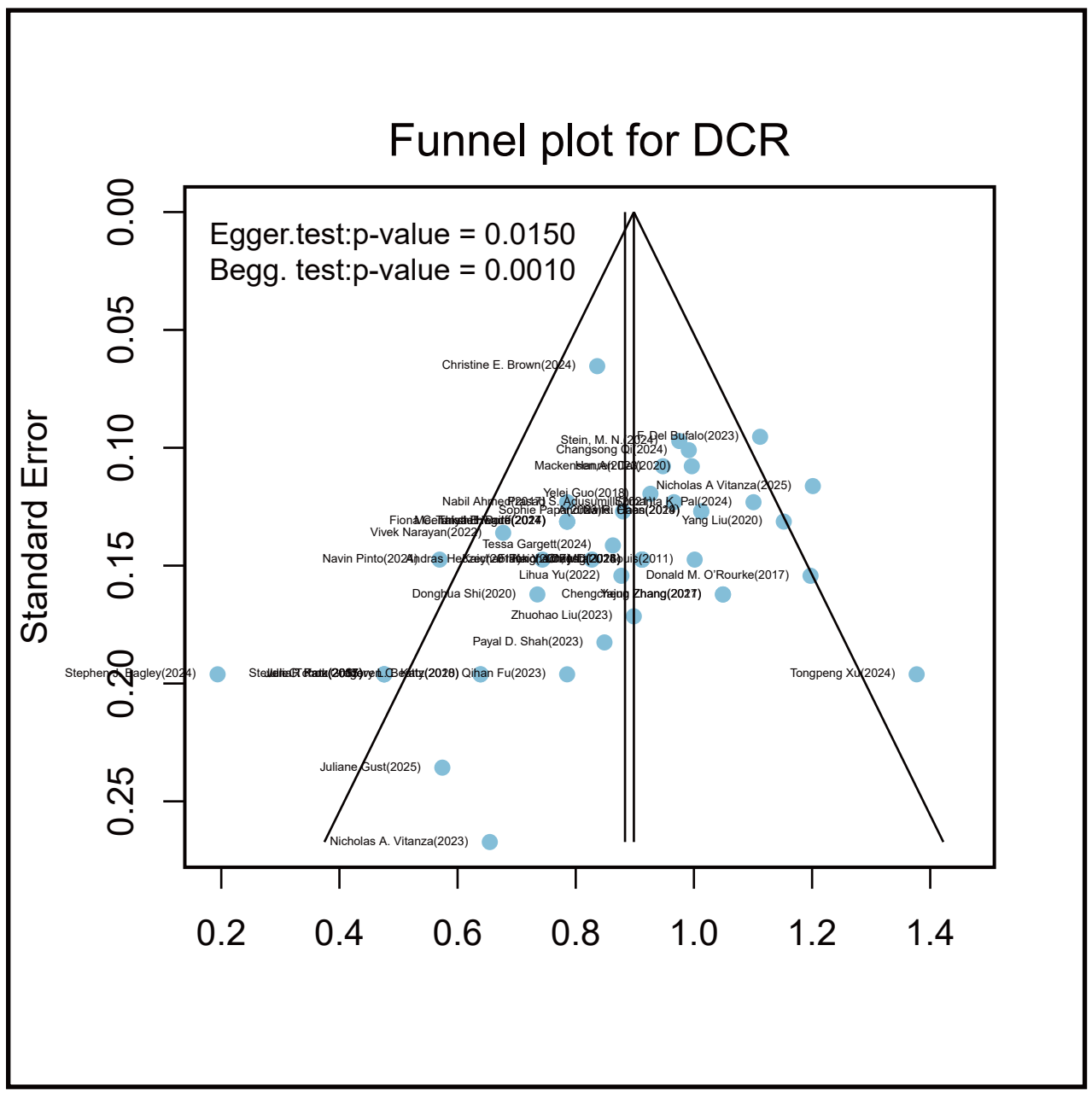

Figure S6. Overall Survival Curves of the (A) stable disease vs. progressive disease group, (B)response vs. no-response group

(A)

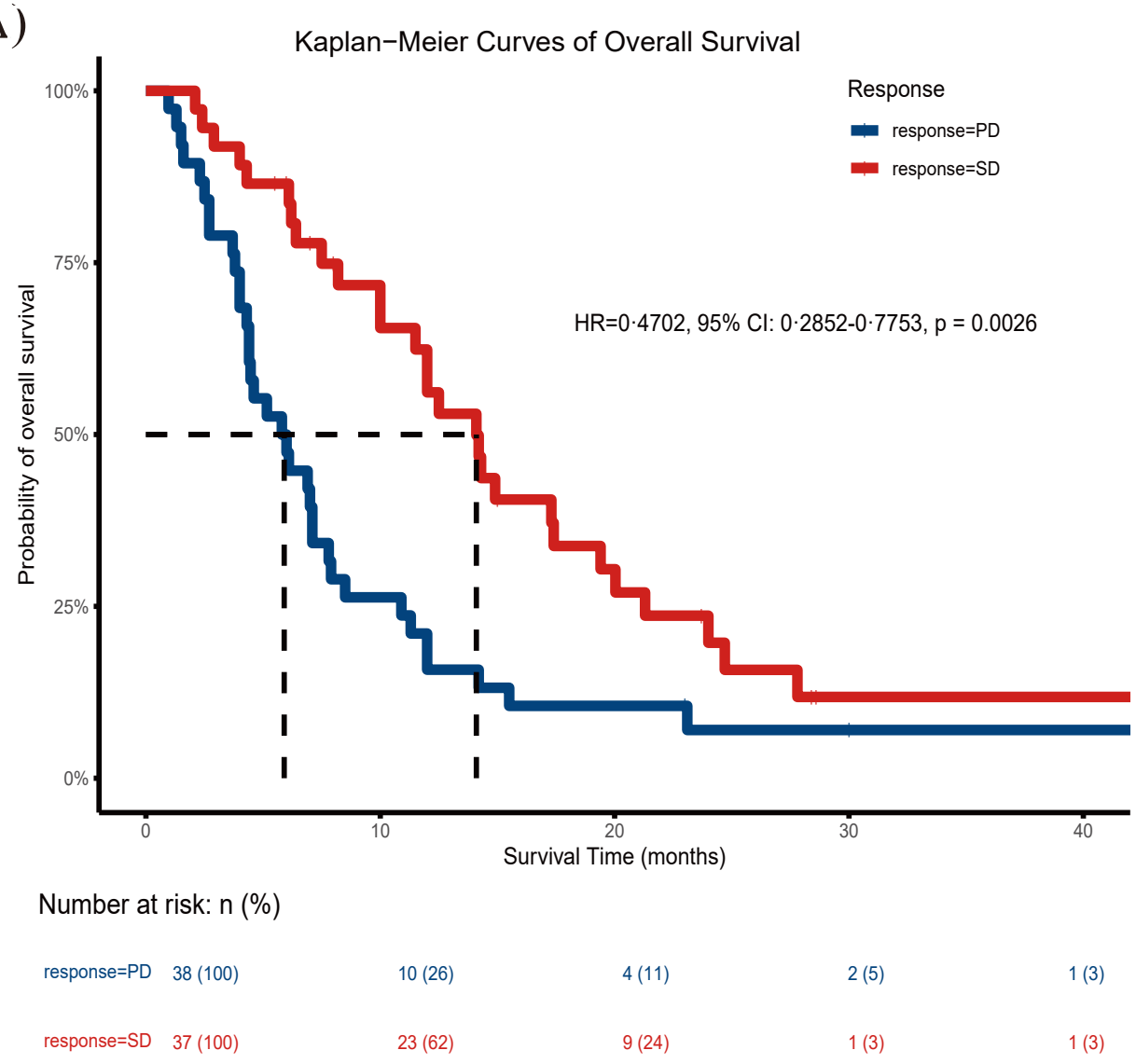

(B)

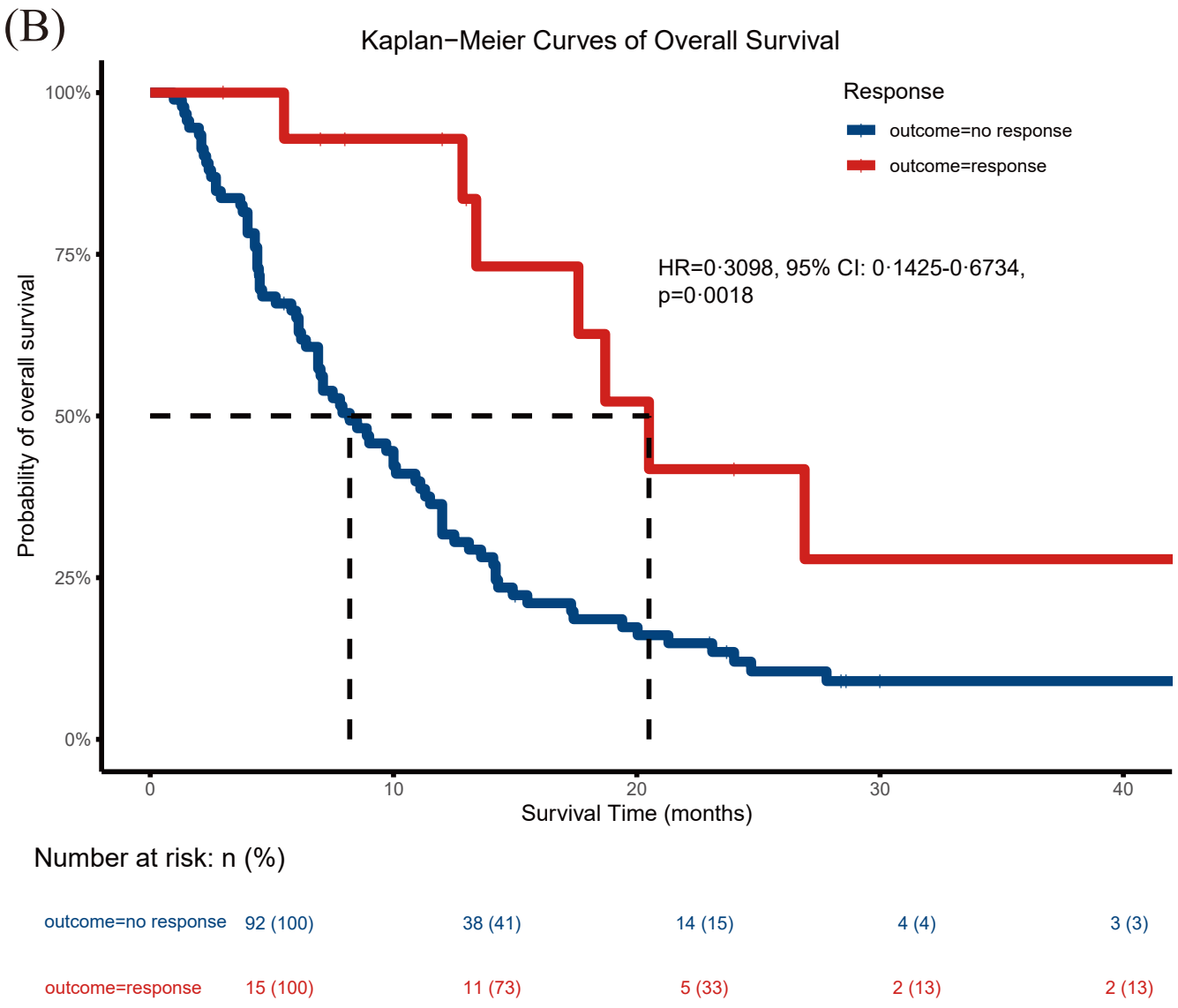

Figure S7. Progression-Free Survival Curves of (A) stable disease vs. progressive disease group, (B)response vs. no-response group,

(A)

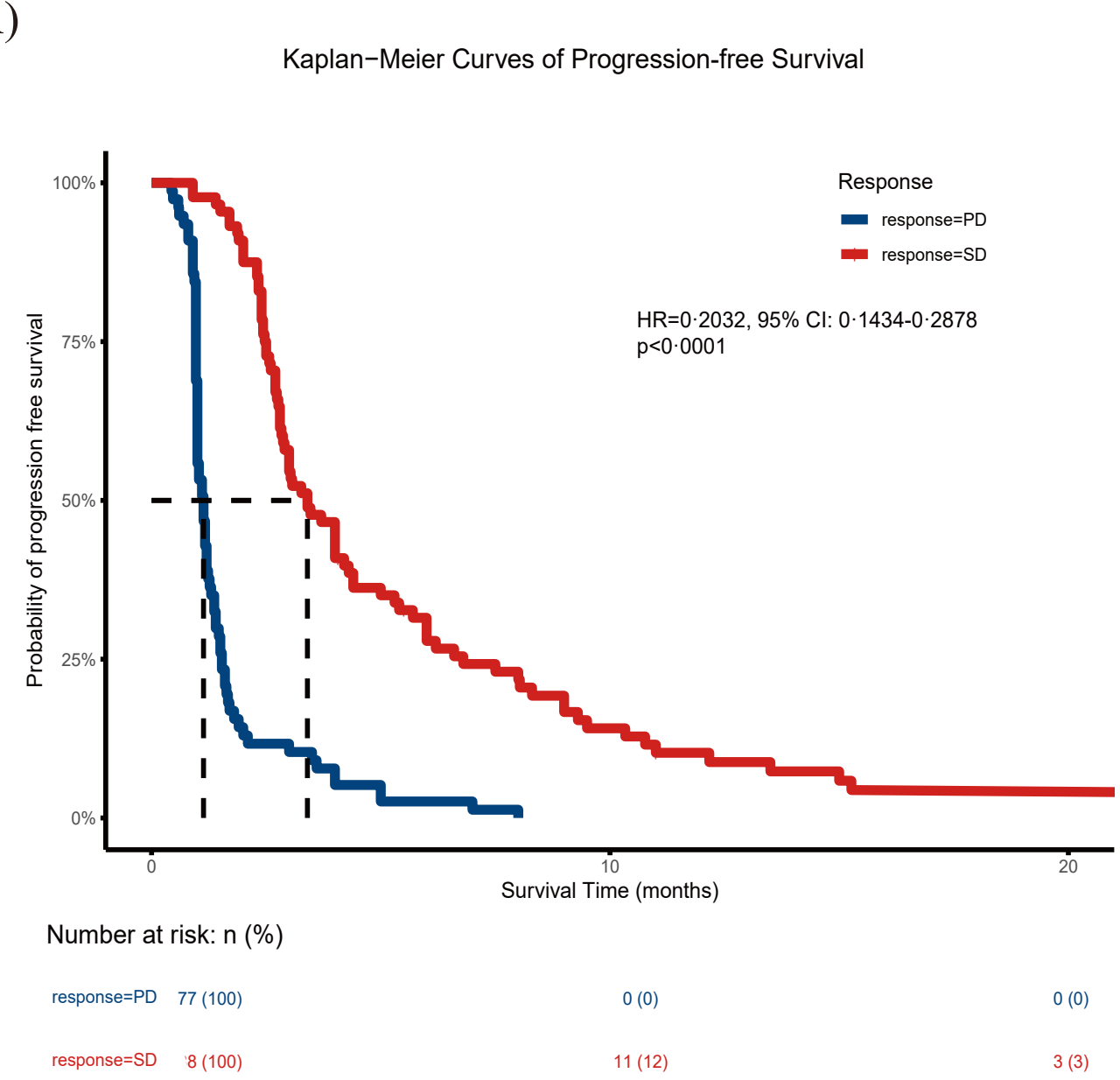

(B)

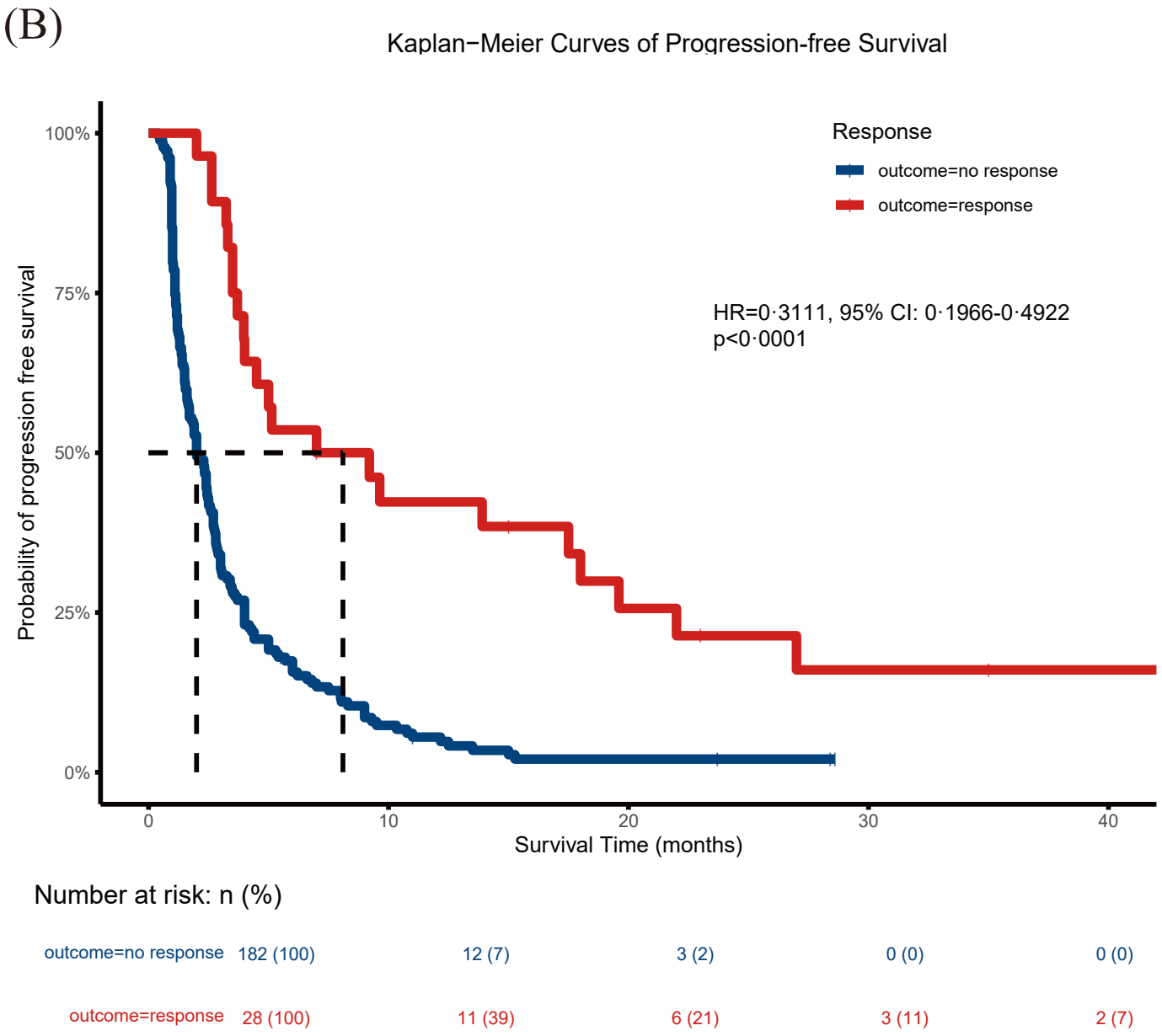

Supplementary Figure 8. Forest plots of the pooled incidence of any-grade adverse events (AEs) and grade ≥3 AEs: (A) Anemia, (B) Neurotoxicity, (C) Respiratory adverse events, (D) Nausea, (E) Vomiting, (F) Cutaneous reaction, (G) Cytokine release syndrome (CRS)

(A)

Gany Anemia	Studies	Patients	Evaluable	Incidence(95%CI)	Grade any Anemia	P_common	P_random
**Subgroup**							
**Lymphdepletion**						< 0.0001***	0.0349*
Yes	14	119	180	0.65 (0.40 − 0.87)			
No	11	20	78	0.30 (0.13 − 0.50)			
**Combination therapy**						< 0.0001***	0.3050
Yes	3	31	36	0.83 (0.16 − 1.00)			
No	22	108	222	0.47 (0.29 − 0.65)			
**Delivery route**						0.0087**	0.1253
Intravenous	15	99	164	0.63 (0.41 − 0.83)			
Regional	10	40	94	0.34 (0.08 − 0.65)			
**Dose frequency**						< 0.0001***	0.2315
Single	12	68	150	0.41 (0.17 − 0.67)			
Multiple	13	71	108	0.63 (0.39 − 0.85)			
Overall Common	25	139	258	0.58 (0.51 − 0.65)			
Overall Random				0.52 (0.33 − 0.70)	0 0.2 0.4 0.6 0.8 1		

G3+Anemia	Studies.	Patients.	Evaluable.	Incidence(95%CI).	Grade 3+ Anemia	P_common.	P_random.
**Subgroup**							
**Lymphdepletion**						0.0547	0.1342
Yes	17	50	268	0.14 (0.04 − 0.27)			
No	11	5	78	0.02 (0.00 − 0.10)			
**Combination therapy**						0.0594	0.1671
Yes	3	8	36	0.20 (0.06 − 0.37)			
No	25	47	310	0.09 (0.02 − 0.19)			
**Delivery route**						< 0.0001***	0.0547
Intravenous	17	45	187	0.16 (0.05 − 0.31)			
Regional	11	10	159	0.03 (0.00 − 0.10)			
**Dose frequency**						0.6460	0.5218
Single	14	24	173	0.08 (0.01 − 0.19)			
Multiple	14	31	173	0.12 (0.01 − 0.29)			
Overall Common	28	55	346	0.08 (0.05 − 0.12)			
Overall Random				0.10 (0.03 − 0.19)	0 0.125 0.25 0.375 0.5		

(B)

Gany Neurotoxicity	Studies	Patients	Evaluable	Incidence(95%CI)	Grade any Neurotoxicity	P_common	P_random
**Subgroup**							
**Lymphodepletion**						< 0.0001***	0.0280*
Yes	9	22	109	0.19 (0.04 − 0.40)			
No	11	55	100	0.62 (0.29 − 0.90)			
**Combination therapy**						0.0051**	0.0045**
Yes	2	0	9	0.00 (0.00 − 0.20)			
No	18	77	200	0.47 (0.25 − 0.70)			
**Delivery route**						0.0027**	0.3437
Intravenous	12	40	133	0.33 (0.09 − 0.62)			
Regional	8	37	76	0.55 (0.20 − 0.88)			
**Dose frequency**						0.3736	0.5225
Single	7	30	93	0.34 (0.10 − 0.63)			
Multiple	13	47	116	0.47 (0.17 − 0.78)			
Overall Common	20	77	209	0.35 (0.28 − 0.42)			
Overall Random				0.42 (0.21 − 0.64)	0 0.2 0.4 0.6 0.8 1		

G3+Neurotoxicity	Studies.	Patients.	Evaluable.	Incidence(95%CI).	Grade 3+ Neurotoxicity	P_common.	P_random.
**Subgroup**							
**Lymphodepletion**						0.0255*	0.0621
Yes	11	10	192	0.02 (0.00 − 0.06)			
No	11	15	100	0.11 (0.02 − 0.25)			
**Combination therapy**						0.6313	0.5585
Yes	2	0	9	0.00 (0.00 − 0.20)			
No	20	25	283	0.05 (0.01 − 0.11)			
**Delivery route**						0.9203	0.9247
Intravenous	13	13	151	0.05 (0.00 − 0.12)			
Regional	9	12	141	0.05 (0.00 − 0.14)			
**Dose frequency**						0.4842	0.8510
Single	8	11	111	0.07 (0.01 − 0.16)			
Multiple	14	14	181	0.04 (0.00 − 0.11)			
Overall Common	22	25	292	0.04 (0.01 − 0.07)			
Overall Random				0.05 (0.01 − 0.10)	0 0.125 0.25 0.375 0.5		

(C)

Gany Respiratory	Studies	Patients	Evaluable	Incidence(95%CI)	Grade any Respiratory	P_common	P_random
**Subgroup**							
**Lymphodepletion**						0.1409	0.2230
Yes	13	50	181	0.26 (0.14 − 0.39)			
No	9	29	80	0.39 (0.21 − 0.59)			
**Combination therapy**						0.9223	0.9233
Yes	4	18	59	0.31 (0.10 − 0.57)			
No	18	61	202	0.30 (0.18 − 0.43)			
**Delivery route**						0.0690	0.3488
Intravenous	14	48	179	0.27 (0.15 − 0.41)			
Regional	8	31	82	0.37 (0.20 − 0.56)			
**Dose frequency**						0.2888	0.6172
Single	11	51	155	0.33 (0.18 − 0.50)			
Multiple	11	28	106	0.27 (0.13 − 0.42)			
Overall Common	22	79	261	0.27 (0.21 − 0.33)			
Overall Random				0.30 (0.20 − 0.42)	0 0.2 0.4 0.6 0.8 1		

G3+ Respiratory	Studies.	Patients.	Evaluable.	Incidence(95%CI).	Grade 3+ Respiratory	P_common.	P_random.
**Subgroup**							
**Lymphodepletion**						0.3990	0.5676
Yes	14	16	199	0.05 (0.02 − 0.11)			
No	9	4	80	0.02 (0.00 − 0.09)			
**Combination therapy**						0.8113	0.8971
Yes	4	4	59	0.04 (0.00 − 0.13)			
No	19	16	220	0.04 (0.01 − 0.09)			
**Delivery route**						0.4903	0.4618
Intravenous	15	16	197	0.05 (0.01 − 0.11)			
Regional	8	4	82	0.01 (0.00 − 0.07)			
**Dose frequency**						0.6378	0.4779
Single	12	11	173	0.04 (0.01 − 0.08)			
Multiple	11	9	106	0.05 (0.00 − 0.14)			
Overall Common	23	20	279	0.04 (0.01 − 0.07)			
Overall Random				0.04 (0.01 − 0.08)	0 0.125 0.25 0.375 0.5		

Supplementary Figure 8. Forest plots of the pooled incidence of any-grade adverse events (AEs) and grade ≥3 AEs: (A) Anemia, (B) Neurotoxicity, (C) Respiratory adverse events, (D) Nausea, (E) Vomiting, (F) Cutaneous reaction, (G) Cytokine release syndrome (CRS)

(D)

Gany Nausea	Studies	Patients	Evaluable	Incidence(95%CI)	Grade any Nausea	P_common	P_random
**Subgroup**							
**Lymphodepletion**						0.6628	0.6154
Yes	13	63	184	0.31 (0.16 − 0.47)			
No	10	24	83	0.38 (0.14 − 0.65)			
**Combination therapy**						0.0801	0.1353
Yes	3	12	55	0.21 (0.10 − 0.33)			
No	20	75	212	0.35 (0.20 − 0.51)			
**Delivery route**						0.0192*	0.8905
Intravenous	13	64	173	0.35 (0.22 − 0.49)			
Regional	10	23	94	0.32 (0.07 − 0.63)			
**Dose frequency**						0.1713	0.6804
Single	14	57	189	0.31 (0.16 − 0.49)			
Multiple	9	30	78	0.37 (0.14 − 0.62)			
Overall Common	23	87	267	0.30 (0.24 − 0.36)			
Overall Random				0.33 (0.20 − 0.47)			
					0 0.2 0.4 0.6 0.8 1		

G3+ Nausea	Studies.	Patients.	Evaluable.	Incidence(95%CI).	Grade 3+ Nausea	P_common.	P_random.
**Subgroup**							
**Lymphodepletion**						0.2475	0.2488
Yes	13	1	184	0.00 (0.00 − 0.02)			
No	10	4	83	0.01 (0.00 − 0.08)			
**Combination therapy**						0.3485	0.3485
Yes	3	0	55	0.00 (0.00 − 0.03)			
No	20	5	212	0.00 (0.00 − 0.02)			
**Delivery route**						0.4149	0.3254
Intravenous	13	1	173	0.00 (0.00 − 0.02)			
Regional	10	4	94	0.01 (0.00 − 0.08)			
**Dose frequency**						0.5555	0.5555
Single	14	4	189	0.00 (0.00 − 0.02)			
Multiple	9	1	78	0.00 (0.00 − 0.04)			
Overall Common	23	5	267	0.00 (0.00 − 0.01)			
Overall Random				0.00 (0.00 − 0.01)			
					0 0.125 0.25 0.375 0.5		

(E)

Gany Vomit	Studies	Patients	Evaluable	Incidence(95%CI)	Grade any Vomit	P_common	P_random
**Subgroup**							
**Lymphodepletion**						0.7309	0.4771
Yes	11	43	135	0.26 (0.10 − 0.44)			
No	9	22	73	0.38 (0.09 − 0.72)			
**Combination therapy**						0.1197	0.1819
Yes	2	5	28	0.11 (0.00 − 0.40)			
No	18	60	180	0.33 (0.16 − 0.52)			
**Delivery route**						0.1529	0.8410
Intravenous	11	46	141	0.30 (0.16 − 0.46)			
Regional	9	19	67	0.33 (0.03 − 0.70)			
**Dose frequency**						0.1552	0.7557
Single	10	35	126	0.28 (0.08 − 0.54)			
Multiple	10	30	82	0.33 (0.11 − 0.58)			
Overall Common	20	65	208	0.27 (0.21 − 0.34)			
Overall Random				0.30 (0.15 − 0.48)			
					0 0.2 0.4 0.6 0.8 1		

G3+ Vomit	Studies.	Patients.	Evaluable.	Incidence(95%CI).	Grade 3+ Vomit	P_common.	P_random.
**Subgroup**							
**Lymphodepletion**						0.1098	0.1550
Yes	11	2	135	0.00 (0.00 − 0.03)			
No	9	7	73	0.05 (0.00 − 0.20)			
**Combination therapy**						0.3701	0.3130
Yes	2	0	28	0.00 (0.00 − 0.05)			
No	18	9	180	0.01 (0.00 − 0.06)			
**Delivery route**						0.0625	0.1273
Intravenous	11	2	141	0.00 (0.00 − 0.02)			
Regional	9	7	67	0.05 (0.00 − 0.21)			
**Dose frequency**						0.2593	0.2748
Single	10	4	126	0.00 (0.00 − 0.03)			
Multiple	10	5	82	0.02 (0.00 − 0.10)			
Overall Common	20	9	208	0.00 (0.00 − 0.03)			
Overall Random				0.01 (0.00 − 0.04)			
					0 0.125 0.25 0.375 0.5		

(F)

Gany Dermatology	Studies	Patients	Evaluable	Incidence(95%CI)	Grade any Dermatology	P_common	P_random
**Subgroup**							
**Lymphodepletion**						0.3519	0.3106
Yes	10	29	135	0.18 (0.08 − 0.30)			
	9	21	80	0.26 (0.13 − 0.42)			
**Combination therapy**						0.4685	0.5232
Yes	1	2	6	0.33 (0.04 − 0.78)			
	18	48	209	0.21 (0.12 − 0.31)			
**Delivery route**						0.0763	0.3485
Intravenous	11	36	137	0.25 (0.16 − 0.36)			
Regional	8	14	78	0.16 (0.03 − 0.33)			
**Dose frequency**						0.3242	0.4852
Single	10	26	99	0.24 (0.13 − 0.38)			
Multiple	9	24	116	0.18 (0.07 − 0.32)			
Overall Common	19	50	215	0.20 (0.14 − 0.26)			
Overall Random				0.21 (0.13 − 0.31)			
					0 0.2 0.4 0.6 0.8 1		

G3+ Dermatology	Studies.	Patients.	Evaluable.	Incidence(95%CI).	Grade 3+ Dermatology	P_common.	P_random.
**Subgroup**							
**Lymphodepletion**						0.4978	0.4869
Yes	10	8	135	0.02 (0.00 − 0.10)			
No	9	1	80	0.00 (0.00 − 0.03)			
**Combination therapy**						0.1633	0.1754
Yes	1	1	6	0.17 (0.00 − 0.64)			
No	18	8	209	0.01 (0.00 − 0.04)			
**Delivery route**						0.4382	0.4319
Intravenous	11	8	137	0.02 (0.00 − 0.09)			
Regional	8	1	78	0.00 (0.00 − 0.03)			
**Dose frequency**						0.6641	0.5986
Single	10	2	99	0.00 (0.00 − 0.04)			
Multiple	9	7	116	0.02 (0.00 − 0.10)			
Overall Common	19	9	215	0.01 (0.00 − 0.03)			
Overall Random				0.01 (0.00 − 0.04)			
					0 0.125 0.25 0.375 0.5		

Supplementary Figure 8. Forest plots of the pooled incidence of any-grade adverse events (AEs) and grade ≥3 AEs: (A) Anemia, (B) Neurotoxicity, (C) Respiratory adverse events, (D) Nausea, (E) Vomiting, (F) Cutaneous reaction, (G) Cytokine release syndrome (CRS)

(G)

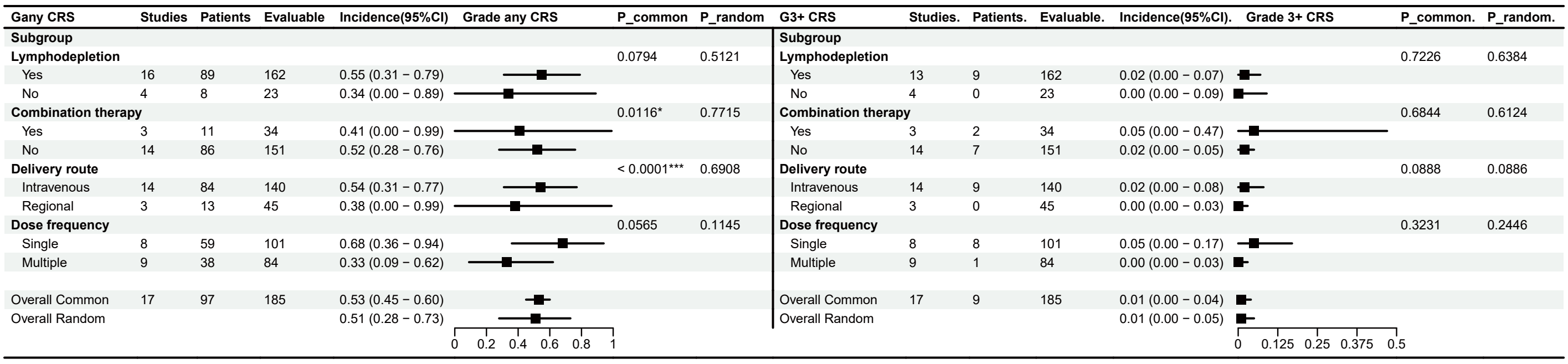

Supplement: Supplementary file 1 [file DataSheet1.pdf]
